# Heterochromatic genome instability and neurodegeneration sharing similarities with Alzheimer’s disease in old *Bmi1*+/− mice

**DOI:** 10.1038/s41598-018-37444-3

**Published:** 2019-01-24

**Authors:** Jida El Hajjar, Wassim Chatoo, Roy Hanna, Patrick Nkanza, Nicolas Tétreault, Yiu Chung Tse, Tak Pan Wong, Mohamed Abdouh, Gilbert Bernier

**Affiliations:** 1 Stem Cell and Developmental Biology Laboratory, Maisonneuve-Rosemont Hospital, 5415 Boul. l’Assomption, Montreal, H1T 2M4 Canada; 2 Department of Psychiatry, McGill University, Montreal, Canada; 3 Douglas Mental Health University Institute, Montreal, Canada; 4 Department of Neurosciences, University of Montreal, Montreal, Canada

## Abstract

Sporadic Alzheimer’s disease (AD) is the most common cause of dementia. However, representative experimental models of AD have remained difficult to produce because of the disease’s uncertain origin. The Polycomb group protein BMI1 regulates chromatin compaction and gene silencing. BMI1 expression is abundant in adult brain neurons but down-regulated in AD brains. We show here that mice lacking one allele of *Bmi1* (*Bmi1*+/−) develop normally but present with age cognitive deficits and neurodegeneration sharing similarities with AD. *Bmi1*+/− mice also transgenic for the *amyloid beta precursor protein* died prematurely and present aggravated disease. Loss of heterochromatin and DNA damage response (DDR) at repetitive DNA sequences were predominant in *Bmi1*+/− mouse neurons and inhibition of the DDR mitigated the amyloid and Tau phenotype. Heterochromatin anomalies and DDR at repetitive DNA sequences were also found in AD brains. Aging *Bmi1*+/− mice may thus represent an interesting model to identify and study novel pathogenic mechanisms related to AD.

## Introduction

Alzheimer’s disease of the late-onset sporadic form (AD) is the most common dementia^1^. At the genetic level, carriers of the *apolipoprotein E4* (*APOE4*) allele have a higher risk to develop the disease with age^2^. Despite this, the etiology of AD remains largely elusive, with old age representing the greatest risk factor. Familial AD (FAD) is an early-onset, autosomal dominant disorder, that is associated with mutations in *amyloid beta precursor protein* (*APP*), *presenilin 1 (PSEN1*) *and presenilin 2 (PSEN2*), and representing less than 5% of all Alzheimer’s disease cases^3^. Short-term memory deficits and behavioral changes are the earliest known manifestations of AD and FAD. While brain atrophy is generally restricted to the limbic system and hippocampus at the disease’s onset, neurodegeneration eventually spreads to all cortical areas, resulting in loss of up to 30% of the brain mass. At the histo-pathological level, the disease is characterized by the accumulation of amyloid plaques and intra-neuronal phospho-Tau (p-Tau) tangles. Early loss of synapses and, later, of neurons, is also a characteristic of the disease^3^. While numerous transgenic mouse models of FAD have been developed that could recapitulate several hallmarks of the disease, experimental mouse models of AD are rare because the disease’s origin remains uncertain^4–6^.

Nucleosomes are the basic building unit of chromatin and are constituted of a 147 base pairs of double-stranded DNA wrapped around a histone octamer^7^. Post-translational modification of histones can modify chromatin compaction and stability. BMI1 (B-cell specific Moloney murine leukemia virus integration site 1) is a core component of the Polycomb Repressive Complex 1 (PRC1). The PRC1 can ubiquitylate histone H2A at lysine 119 (H2A^ub^) through the E3-ubiquitin ligase activity of Ring1a/b, its catalytic unit^8–11^. The main classical function of BMI1 is to maintain chromatin compaction and gene silencing at developmental and senescence-associated loci. The *Ink4a* locus, which encodes for the p16^Ink4a^ and p19^Arf^ tumor suppressor proteins, is one of the main targets of BMI1 repressive activity, and cells deficient for *Bmi1* undergo rapid senescence^12,13^. *Bmi1*-null mice present axial skeleton defects, reduced post-natal growth and lifespan, as well as cerebellar degeneration^14^. The BMI1 protein is also recruited at DNA break sites where it can promote DNA damage response (DDR) and repair^15,16^. *Bmi1*-deficiency in mice is furthermore associated with accumulation of DNA damage and reactive oxygen species (ROS), both of which can be improved through co-deletion of *Chk2* or *p53*^17,18^. More recently, BMI1 was found to be abundant at heterochromatin regions containing repetitive DNA sequences (namely the constitutive heterochromatin). *BMI1* knockdown resulted in reduced histone H3 trimethylation at lysine 9 (H3K9^me3^) and in transcriptional activation of tandem repeats in mammalian cells^19^.

We recently reported that the expression of *BMI1* is reduced in AD brains and in AD neurons produced from the differentiation of patient-specific induced pluripotent stem cells. Notably, acute *BMI1* inactivation in mature human neurons could recapitulate most AD-related neuronal pathologies^20^. Herein, we show that mice lacking one *Bmi1* allele (*Bmi1*^+/−^) develop normally, but display with age a pathology sharing some similarities with AD. Crosses between *Bmi1*^+/−^ mice and transgenic mice carrying a mutant human APP gene revealed genetic cooperation in disease onset and severity. Furthermore, loss of heterochromatin and DDR at repetitive DNA sequences in neurons was characteristic of *Bmi1*^+/−^ mice and AD brains. Experimental evidences in mice furthermore suggested a significant contribution of the DDR to the observed neuronal pathology. Loss of one *Bmi1* allele in mice thus predisposes to age-related neurodegeneration sharing some similarities with AD.

## Methods

### Human samples

Paraffin-embedded human brains were obtained from the department of pathology of Maisonneuve-Rosemont Hospital. Frozen post-mortem human cortices are a gift from the Douglas Hospital Brain Bank. The human samples were used accordingly to the Maisonneuve-Rosemont Hospital Ethic Committee (Approval ID #2012-481, 11065). All samples were obtained after informed consent of the patients. Samples were confirmed by histo-pathological analysis as non-demented controls, FAD or AD cases. The list of human samples is described in Supplemental Table 1.

### Animals

Mice (male and female) were used in compliance with- and with the approval of the Animal Care Committee of the Maisonneuve-Rosemont Hospital Research Center (Approval ID #2009-40; #2009-42; # 2011-23). The FAD mouse model (Tg-Thy-1APPSwDutIowa in C57BL/6 J background) was purchased from The Jackson laboratory^21^. *Chk2*^+/−^ mice were obtained from N. Motoyama. *p53*^+/−^ and *Ink4a*^+/−^ mice were obtained from The Jackson Laboratory^17,22^. *Bmi1*^+/−^ mice were a generous gift from M. van Lohuizen (Netherlands)^14^. All mice were maintained in the C57BL/6 J genetic background. *Bmi1*^+/−^ mice were crossed with either *Chk2*^+/−^ or *p53*^+/−^ mice, to obtain *Bmi1*^+/−^*/Chk2*^+/−^ or *Bmi1*^+/−^*/p53*^+/−^ mice. *Bmi1*^+/−^*/Chk2*^+/−^ or *Bmi1*^+/−^*/p53*^+/−^ mice were then intercrossed to obtain *Bmi1*^−/−^*/Chk2*^−/−^ or *Bmi1*^−/−^*/p53*^−/−^ mice. From several breeding, we obtained the expected Mendelian ratio for *Bmi1*^−/−^*/Chk2*^−/−^ mice (12.5%), but not for *Bmi1*^−/−^*/p53*^−/−^ mice (1 animal out of 200 offspring or 0.5%), suggesting that the *Bmi1/p53* double mutant is lethal^23^. A similar strategy was used to generate *Bmi1*^−/−^*/Ink4a*^−/−^ mouse embryos in order to perform the neuronal cultures. Wild type (*Bmi1*^+/+^) and *Bmi1*^−/−^ animals used for comparison were littermates of the double mutants.

### Murine neuronal cultures

After dissociation of E18.5 cortices, cells were plated at 1.5 × 10^5^ cells/well on poly-L-lysine-coated 8-well cultures slides (BD Biosciences). Cells were maintained in normal medium composed of Neurobasal-A Medium (Invitrogen), Glutamax-I (Gibco), gentamycin (50 µg/ml; Gibco), B27 supplement (Gibco), nerve growth factor (50 ng/ml; Invitrogen) and BDNF (0,5 ng/ml; Invitrogen). For the assays using inhibitors, we added 2 μM of DMSO, 2 μM of ATM/ATR inhibitor (CGK733; Millipore) or 10 μM of ATM inhibitor (KU55933; KuDOS Pharmaceuticals) to the cell culture medium for 16 hours. NAC was added daily at 5 mM into the culture medium for 7 days^18^.

### Amyloid fractions and Immunoprecipitation

Cortices were minced and homogenized in a 10 ml TBS 1 × (50 mM Tris-Cl, pH 7.5. 150 mM NaCl)/protease inhibitor cocktail solution (Roche Diagnostics), using a tissue grinder. The homogenates were sonicated and ultra-centrifuged at 175,000 g for 1 h at 4 °C. The supernatants were collected as the soluble cellular fraction^24^. The dense pellet was re-homogenized in a 10 ml TBS 1×/1% Triton X-100/protease inhibitor cocktail solution, followed by sonication and centrifugation at 100,000 g for 1 h at 4 °C. The supernatants were recuperated as the insoluble cellular fraction^24^. For immunoprecipitation, protein samples and cellular fractions were incubated overnight with the primary antibody followed by incubation with the protein A/G beads (Millipore) for 2 hours the second day. Beads with bound immunocomplexes were washed with RIPA buffer (50 mM Tris-HCl, pH 7.5; 150 mM NaCl; 0.1%Tween 20; protease inhibitors Complete) and bound proteins were subsequently heat eluted with 1x Laemmli buffer.

### Western blot

Cortices were minced and homogenized in 5 ml of complete mini protease inhibitor cocktail solution (100 mM Tris-HCl, pH7.5; 150 mM NaCl; 0.1% Tween 20; 2% SDS; protease inhibitors Complete; Roche Diagnostics) using a tissue grinder. The homogenates were sonicated and quantified using the Bradford reagent. For Western Blot, proteins were resolved by SDS-PAGE electrophoresis and transferred to a nitrocellulose blotting membrane (Bio-Rad). Membranes were incubated with the primary antibodies, treated with the corresponding horseradish peroxidase-conjugated secondary antibodies (Sigma), and developed using the Immobilon Western (Millipore).

### Immunohistochemistry

Tissues were immersed in Formalin or 4% paraformaldehyde/3% sucrose in 0.1 M phosphate buffer, pH 7.4 for 16 h at room temperature on a rocking platform. Samples were washed three times in PBS and embedded in paraffin according to standard protocols. 8 µm thick sections were mounted on Super-Frost glass slides (Fisher Scientific) and processed for immunohistochemistry staining. Paraffin-embedded slices were analyzed by using the Vectastain^®^ ABC kit (Vector) according to the manufacturer instructions. Peroxidase substrates used are the Vector^®^ VIP (Violet) (Vector), and DAB (brown) (Sigma)^18^. Observations were made using the Zeiss imager Z2 microscope and images were captured with a digital camera. To obtain comparable results, we selected pyramidal neurons from the frontal cortex to quantify the number of chromocenter/neuron. Pyramidal neuron identity was determined by the expression of NeuN, the cell morphology (using phase contrast imaging), the large nuclear diameter (when compared to astrocytes), and the presence of chromocenters and of a large nucleolus.

### Senescence-associated β-galactosidase assay

Senescence-associated (SA) β-galactosidase staining was detected histochemically at pH 6 as described previously^25^. Briefly, tissue slices were fixed in PBS containing 1% formaldehyde, 0.4% glutaraldehyde, and 0.02% Igepal. After three washes, slices were exposed to the X-gal solution (1 mg/ml X-gal; 5 mM K3Fe(CN)6; 5 mM K4Fe(CN)6; 1 mM MgCl2, in PBS; pH 6.0). Slides were mounted and observations made under a microscope.

### Antibodies

Rabbit anti-p53 (Santa Cruz Biotechnology), rabbit anti-p-JNK (Invitrogen), mouse anti-synaptophysin (Sigma), mouse anti-amyloid clone DE2B4 (Abcam), rabbit anti-amyloid clone FCA3542 (Calbiochem), mouse anti-amyloid MOAB2 (Novus; NBP2-13075)), pan anti-Tau k9JA (DAKO); mouse anti-p-Tau clone AT-8 (Thermo scientific), mouse anti-p-Tau clone PHF1 (a gift from Dr. Davies, Albert Einstein College of Medicine), mouse anti-NeuN (Chemicon), rabbit anti-cleaved caspase-3 (Cell Signaling), rabbit anti-GFAP (Dako; Z0334), Iba1 (Wako; 019-19741), mouse anti-Bmi1 clone F6 (Millipore), mouse anti-Bmi1 (Abcam), mouse anti-p-ATM (Novus), rabbit anti-p-ATR (Santa Cruz Biotechnology), rabbit anti-H3K9^me3^ (Abcam), mouse anti-H2Aub clone E6C5 (Millipore), mouse anti-HP1 (Millipore), mouse anti-β-actin (Sigma), mouse anti-tubulin (Sigma), mouse anti-H3 (Upstate), and rabbit anti-mouse IgG (Upstate).

### RT and real-time PCR

All primers were designed to flank individual exons and tested by PCR in RT+ and RT− control extracts. Total RNA was isolated using TRIzol reagent (Invitrogen). The RNA was treated with DNaseI for 20 min prior to reverse transcription (RT) and PCR for satellite repeats, which are intron-less. Reverse transcription was performed using 1 µg of total RNA and the MML-V reverse transcriptase (Invitrogen). Real-time PCR was performed using the Platinum SYBRGreen SuperMix (Invitrogen) and a Real-Time PCR apparatus (BioRad). Primers sets used were as follow: p16Ink4a (F) 5_-CAACGCCCCGAACTCTTTC-3_; (R) 5_-GCAGAAGAGCTGCTACGTGAAC-3_, p19Arf (F) 5_-GGCTAGAGAGGATCTTGAGAAGAGG-3_; (R) 5_-GCCCATCATCATCACCTGGTCCAGG-3_. Primers sets used for Bmi1, IAP, Maj Sat, Min Sat, Line, Sine and IAP were as described^19^.

### Chromatin Immunoprecipitaion (ChIP) assay

ChIP was performed using the ChIP Assay kit (Upstate). Briefly, 50 mg of cortical tissue was frozen for 1 hour and then homogenized at RT according to the manufacturer’s protocol. The tissue was sonicated on ice for 10 sec at 30% amplitude to shear the chromatin (Branson Digital Sonifier 450, Crystal Electronics, On. Canada). Sonicated materials were immunoprecipitated using designated antibodies. Fragments were then amplified by real-time PCR in triplicates. Human primers sets used were as follow: MCBOX (F) 5′-AGGGAATGTCTTCCCATAAAAACT-3′; (R) 5′-GTCTACCTTTTATTTGAATTCCCG-3′; SATIII (F) 5′-AATCAACCCGAGTGCAATCNGAATGG-3′; (R) 5′-TCCATTCCATTCCTGTACTCGG-3′; SATa (F) 5′-AAGGTCAATGGCAGAAAAGAA-3′; (R) 5′-CAACGAAGGCCACAAGATGTC-3′; ACTIN (F) 5′-CCTCAATCTCGCTCTCGCTC-3′; (R) 5′-CTCTAAGGCTGCTCAATGTCA-3′; ß-GLOBIN (F) 5′-GGCTGTCATCACTTAGACCTC-3′; (R) 5′-GGTTGCTAGTGAACACAGTTG-3′;5′. Mouse primers sets used were as follow: MajSAT 5′- GGCGAGAAAACTGAAAATCACG-3′, 5′-CTTGCCATATTCCACGTCCT-3′; MinSAT 5′- TTGGAAACGGGATTTGTAGA-3′, 5′-CGGTTTCCAACATATGTGTTTT-3′; LINE 5′-TGGCTTGTGCTGTAAGATCG-3′, 5′-TCTGTTGGTGGTCTTTTTGTC-3′; SINE1 5′-GAGCACACCCATGCACATAC-3′, 5′-AAAGGCATGCACCTCTACCACC-3′; Actin 5′-TCGATATCCACGTGACATCCA-3′; 5′-GCAGCATTTTTTTACCCCCTC-3′; HoxA7 5′-GTGGGCAAAGAGTGGATTTC-3′; 5′-CCCCGACAACCTCATACCTA-3′; β-major 5′-CAGTGAGTGGCACAGCATCC-3′; 5′-CAGTCAGGTGCACCATGATGT-3′. ChIP-qPCR data was analyzed according to the Percent Input method^19^. First, the raw Ct of the diluted 1% input fraction is adjusted by subtracting 6.64 cycles (i.e. log2 of the dilution factor 100). Subsequently, the percent input of each IP fraction is calculated according to this equation: 100*2^(Adjusted Input Ct-Ct(IP)^.

### Behavioral tests

Behavioral assays were performed at the Douglas Hospital Institution, Montreal, Canada under the supervision of Dr. Rocheford. Male mice were first accustomed to the test environment prior to testing. Groups of 15-month old male WT (n = 8) and *Bmi1*^+/−^ (n = 8) mice were tested successively and were habituated, trained, and evaluated at the same time of day for each experiment. Behavioral tests included Morris water maze test and Barnes maze assay. In the Morris water maze assay, a hidden escape platform is immersed just below water level with visual cues (color) around the pool in plain eyesight of the mouse. Upon release, the mouse navigates around the pool seeking an exit while various parameters are recorded, including the time spent in each quadrant of the pool and the time taken to reach the platform (latency). Barnes maze assay includes a circular platform containing 20 circular holes around its circumference and visual cues (colored shapes) surrounding the surface in plain eyesight of the mouse. The platform is intensely lit by an overhead lamp, and underneath one of the holes there is an exit box. Different parameters are analyzed comprising latency to escape and number of errors.

### LTP assay

For this study, we used 15-month old male WT and *Bmi1*^+/−^ mice. Under deep anesthesia, brains were removed and coronal brain slices (350 µm thickness) containing the hippocampus were cut in hyperosmotic, ice-cold and carbogenated (bubbled by 95%O_2_/5%CO_2_ to maintain the pH at 7.4) slice cutting solution (in mM: 252 sucrose, 2.5 KCl, 4 MgCl_2_, 0.1 CaCl_2_, 1.25 KH_2_PO_4_, 26 NaHCO_3_ and 10 glucose) using a Vibratome. Freshly cut slices were placed in an incubating chamber with carbogenated artificial cerebrospinal fluid (ACSF (~310 mOsmol/L) consists of (mM): 125 NaCl, 2.5 KCl, 1 MgCl_2_, 2 CaCl_2_, 1.25 NaH_2_PO_4_, 26 NaHCO_3_ and 25 glucose). Slices were recovered at 32 °C for one hour and subsequently maintained at room temperature. Carbogenated ACSF containing bicuculline methbromide (5 µM) to block GABA_A_ receptor-mediated inhibitory synaptic current was used to perfuse slices in all recordings. Postsynaptic responses, which were evoked by stimulating the Schaffer collateral-commissural pathway via constant current pulses (0.08 ms) delivered through a tungsten bipolar electrode (FHC), were recorded from the hippocampal CA1 region, amplified by a Multiclamp 700B (Axon) and stored in a PC for offline analysis using Clampfit (Axon). All recordings were performed at room temperature. Field excitatory postsynaptic potential (fEPSP) was evoked at 0.05 Hz and detected by an ACSF-filled glass electrode that was placed in the stratum radiatum of the hippocampal CA1 region. Long-term potentiation (LTP) of fEPSP was induced by a high-frequency tetanus (100 Hz, 100 pulses). Student’s t-test was used to compare changes in fEPSP slope at 60 minutes after tetanus between WT and *Bmi1*^+/−^ groups. In order to determine if fEPSPs are potentiated by tetanus within each mouse group, paired Student’s t-test was used to compare fEPSP slope recorded before (baseline) and at 60 minutes after tetanus.

### Statistical analysis

The Student’s *t*-test for unpaired samples was used to measure statistical differences. An analysis of variance (ANOVA) followed by the Dunnett test was used for multiple comparisons with one control group. In each figures, the criterion for significance (*P* value) was mentioned.

## Results

### *Bmi1*^+/−^ mice develop accelerated aging features and neurological symptoms

**Figure 1:**
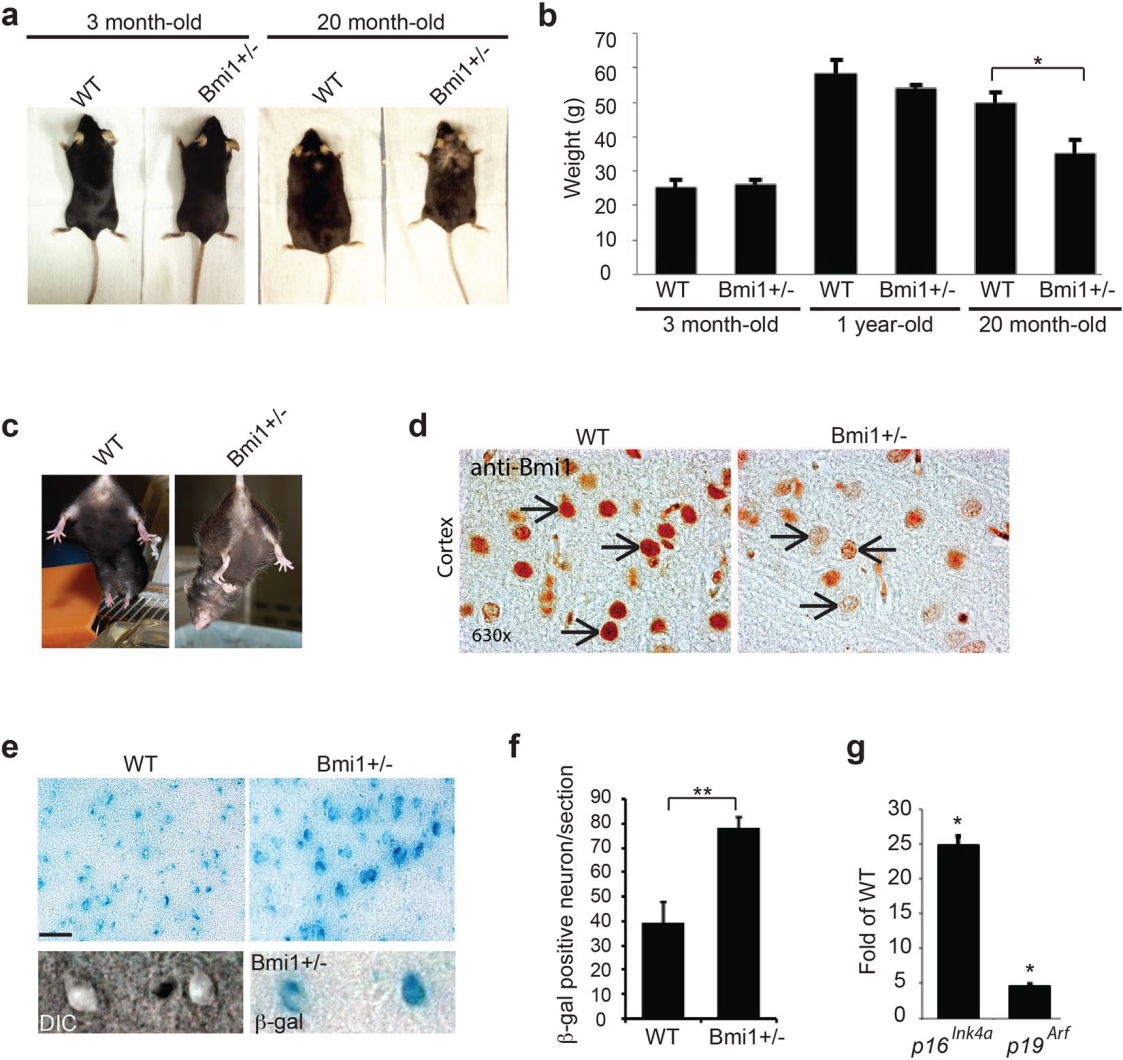
*Bmi1*^+/−^ mice present accelerated aging features and neurological symptoms. (**a**) Photographs of 3 month-old and 20 month-old WT and *Bmi1*^+/−^ mice. Note that aged *Bmi1*^+/−^ mice are smaller and exhibit hair loss (alopecia) compared to WT littermates (n = 5). (**b**) Quantification representing the mean weight at 3 months (n = 6), 1-yr (n = 6) and 20 months (n = 6) of WT and *Bmi1*^+/−^ mice. (**c**) Photographs showing the paw clasping reflex of aged WT (n = 5) and *Bmi1*^+/−^ mice (n = 5). Note that the *Bmi1*^+/−^ mouse pulls its fore limbs into its body rather than spreading them away from its trunk, and present an important asymmetry in the hind limbs’ position. (**d**) IHC analysis on mouse cortical sections showing robust Bmi1 expression in the nucleus of WT neurons (arrows) and weak expression in *Bmi1*^+/−^ neurons (arrows). (**e**) Analysis of SA β-galactosidase activity in the cortices of old WT and *Bmi1*^+/−^ mice. Scale bar: 20μm. (**f**) Quantification of the number of SA β-galactosidase positive neurons/cortical section. (**h**) Quantitative PCR analysis of *p16*^*Ink4a*^ and p19^Arf^ expression in the cortices of old WT (n = 3) and *Bmi1*^+/−^ (n = 3) mice.

We previously reported that *Bmi1*^+/−^ mice have reduced median and maximal lifespan along with apparition of lens cataracts at higher frequencies than WT littermates, suggesting that *Bmi1* hemi-deficiency may accelerate the aging process^18^. Alopecia and weight loss are common characteristics of premature aging in mice^26–30^. We observed that between the ages of 15–24 months (defined here as *old* mice), *Bmi1*^+/−^ mice exhibited alopecia and reduced body size (~25%) when compared with age-match WT littermates (Fig. 1a,b). These pathological features were not present in 3-month and 1-year old *Bmi1*^+/−^ mice (Fig. 1a,b). We performed the paw-clasping test to verify if old *Bmi1*^+/−^ mice presented pathological reflexes. Adult WT mice picked by the tail and slowly inclined towards a horizontal surface spread all four limbs in anticipation of contact. However, instead of limb extension, old *Bmi1*^+/−^ mice displayed an irregular clasping behavior characterized by the dragging of the fore paws towards the trunk and asymmetry in limb position (Fig. 1c). This phenotype is generally associated with a neurological syndrome^31^.

The Bmi1 gene operates as a central regulator of apoptosis and senescence through repression of the p19^Arf^/p53 and p16^Ink4a^/pRb tumor suppressor axes. Senescence is usually excluded in post-mitotic cells such as neurons, yet increased SA β-galactosidase activity and expression of the senescence-associated genes *p19*^*Arf*^ and *p16*^*Ink4*^ have been noted in cortical neurons of aged *p73*^+/−^ mice and of *Bmi1*^−/−^ pups^18,25,32^. Furthermore, expression of *p16*^*Ink4*^ is considered as a biomarker of aging^33^. We first confirmed neuronal expression of Bmi1 in the cortex of old WT mice and its severe reduction in old *Bmi1*^+/−^ mice (Fig. 1d). Next, we compared these old WT and *Bmi1*^+/−^ mice for SA β-galactosidase activity and *p16*^*Ink4*^/*p19*^*Arf*^ expression. We observed robust SA β-galactosidase staining in pyramidal neurons and a 2-fold increase in the total number of SA β-galactosidase-positive neurons/section in layers 2 and 3 of the frontal cortex of *Bmi1*^+/−^ mice (Fig. 1e,f). This was accompanied with significant increased in *p16*^*Ink4*^ and *p19*^*Arf*^ expression (Fig. 1g). These findings suggested that in addition to affect lifespan, *Bmi1* hemi-deficiency resulted in the apparition of an age-dependent neurological syndrome associated with neuronal senescence.

### *Bmi1*^+/−^ mice show altered spatial memory and reduced long-term potentiation

**Figure 2:**
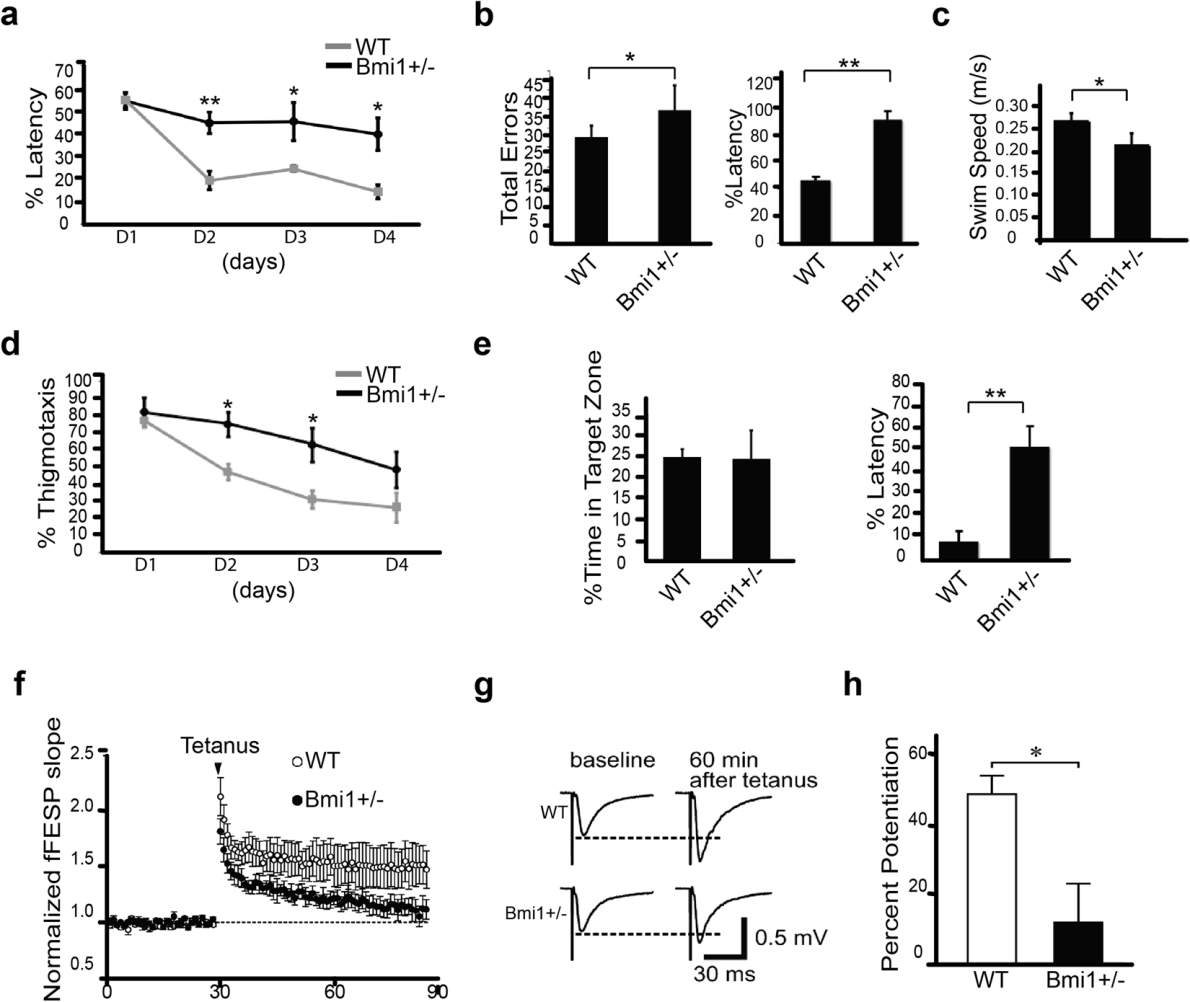
*Bmi1*^+/−^ mice exhibit memory and LPT impairment. (**a**–**e**) 15-month old male WT (n = 8) and *Bmi1*^+/−^ (n = 8) mice were tested for behavioral changes associated with spatial memory formation deficit. (**a**–**c**) Results of Morris water maze probe test, showing time spent in the target quadrant searching for the hidden platform, mean time to reach the target platform (latency) and mean swim speed. (**d**) Charts representing mean thigmotaxis of Morris water maze 4-day trials. (**e**) Histograms representative of Barnes maze test results including mean total errors and mean latency to reach the target probe. (**f**–**h**) LTP assays on hippocampal slices of 15-month old male WT and *Bmi1*^+/−^ mice. (**f**) Scatter plots revealed changes in the slope of field excitatory postsynaptic potential (fEPSP) recorded from the hippocampal CA1 region of WT and *Bmi1*^+/−^ mice. LTP was induced by tetanus (100 Hz, 100 stimulation). Dotted line represents the baseline. (**g**) Representative traces of fEPSP recorded before (baseline) and 60 min after tetanus. Dotted lines represent baselines. (**h**) Histogram of fEPSP slope at 60 min after LTP induction. Normalized fEPSP slopes, recorded between 55–60 min after tetanus, were averaged for calculating percent potentiation. Values are mean ± SEM. *P < 0.05; **P < 0.01; Student’s t-test.

To test whether the neurological symptoms were associated with perturbed cognitive functions, we examined apparently healthy 15 month-old WT and *Bmi1*^+/−^ mice for spatial learning and memory aptitude using the Morris water and Barnes maze tests. For the Morris water navigation task, mice were trained for 4 days to locate the platform (training) and then evaluated on their capacity to remember the platform location (probe test). *Bmi1*^+/−^ mice performed poorly in this assay during both the training period and probe test, as demonstrated by the increased time (latency) required to reach the platform (Fig. 2a,b). *Bmi1*^+/−^ mice also exhibited an anxious phenotype as demonstrated by an increased thigmotactic behavior while performing the training (Fig. 2d)^34^. The swimming speed of aged *Bmi1*^*+/−*^ mice was also reduced (0.25 m/s for WT and 0.20 m/s for *Bmi1*^+/−^) (Fig. 2c). In contrast, the swimming speed of 1 year-old WT and *Bmi1*^+/−^ mice was nearly identical (not shown), thus consistent with the apparition of a progressive, age-related reduction in locomotors strength. The Barnes maze test is based on mice aversion to open enlightened spaces, which ultimately prompts them to find secure shelter. *Bmi1*^+/−^ mice were more prone to visiting non-target holes while searching for the hole with the escape box and the latency of escape was also increased (Fig. 2e). Although these results suggested impaired spatial memory formation, the reduction in swimming speed and the possibility of visual dysfunction^23^ led us to perform electrophysiological analyses to further test this possibility.

LTP is a cellular surrogate for spatial memory formation^35^. Using the same animals as in the behavioral tests, LTP of the Schaffer collateral pathway was induced in the CA1 region of the hippocampus. We found that a commonly used protocol for inducing LTP (100 Hz, 100 pulses) enhanced fEPSP slope in WT mice at 60 minutes after tetanus (Percent potentiation: 48.9 ± 5.2%, data from 6 slices and 3 mice). Compared with fEPSP recorded before tetanus (baseline), WT slices displayed significant potentiation (*P* = 0.036; paired Student’s t-test) (Fig. 2f–h). Slices from *Bmi1*^+/−^ mice exhibited weaker potentiation than WT (11.7 ± 11.2%, data from 6 slices, 4 mice; transgenic vs. WT: *P* = 0.045, Student’s t-test) and failed to show significant potentiation when compared to baseline at 60 minutes after tetanus (*P* = 0.357; paired Student’s t-test) (Fig. 2f–h). Our findings demonstrated that LTP formation in *Bmi1* hemi-deficient mice was significantly impaired, thus strengthening the hypothesis of impaired spatial memory formation.

### Old *Bmi1*^+/−^ mice present a pathology showing some features of AD

**Figure 3:**
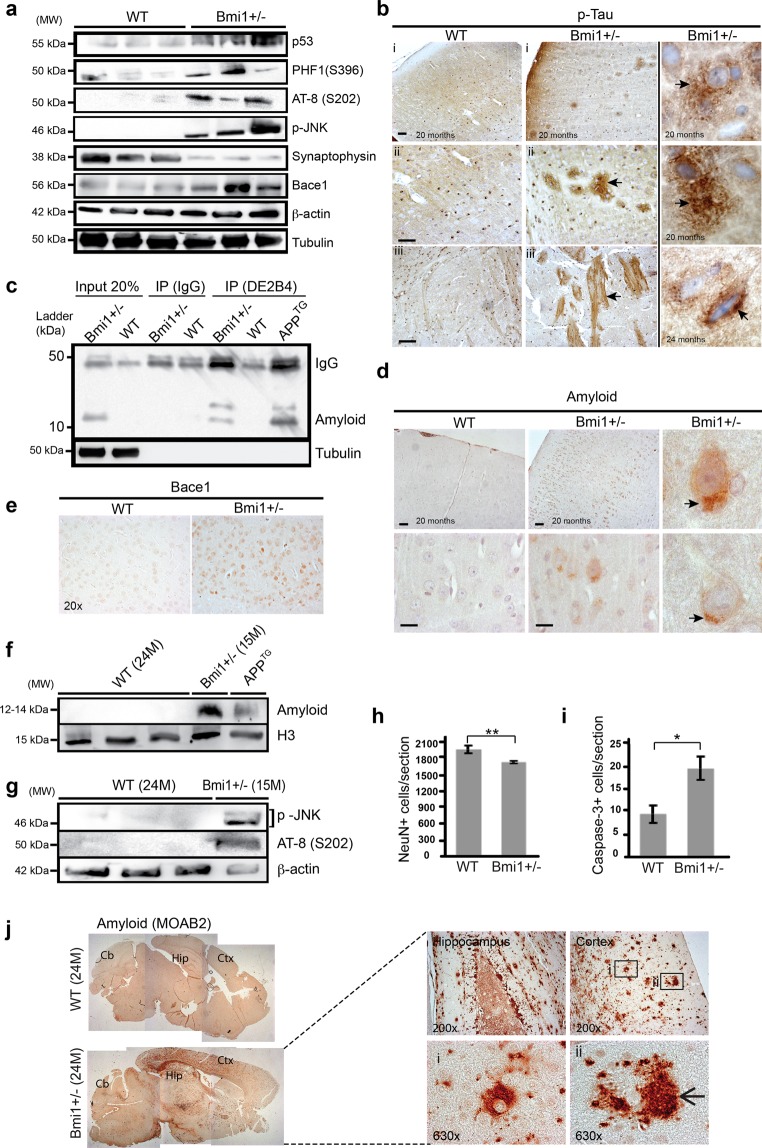
Old *Bmi1*^+/−^ mice present cortical neurodegeneration. (**a**) Western blot analysis on cortical extracts from 15–20 month-old WT and *Bmi1*^+/−^ mice. (**b**) IHC for p-Tau (PHF1) on cortical sections of old WT (n = 4) and *Bmi1*^+/−^ (n = 4) mice. (i) Low magnification. Scale bar: 20μm. Higher magnifications in (ii) revealed p-Tau deposits on ghost-like neurons and in (iii) p-Tau immunoreactivity in track fibers of the cortical white matter of *Bmi1*^+/−^ mice. The 3 insets show images of the most severe cases observed of p-Tau accumulation in *Bmi1*^+/−^ mouse neurons (arrows). Scale bar: 8μm. (**c**) Immuno-precipitation (IP) of the β-amyloid peptide (DE2B4 antibody) using the soluble cellular fraction of cortices from old WT and *Bmi1*^+/−^ mice, and from a 6-month old *APP* transgenic mouse (positive control). The blot was revealed using the FCA3542 antibody. N = 2 independent experiments. (**d**) IHC on brain sections of old WT (n = 4) and *Bmi1*^+/−^ (n = 4) mice for the expression of β-amyloid (DE2B4 antibody). Top images scale bar is 20μm; Lower images scale bar is 8μm. Arrows in the insets indicate amyloid accumulation in the neuronal cell body. (**e**) IHC staining for Bace1 on brain sections of old WT (n = 2) and *Bmi1*^+/−^ (n = 2) mice. (**f**,**g**) Immuno-blot analysis of cortical extracts from 24 month-old WT and 15 month-old *Bmi1*^+/−^ mice showing accumulation of pathological proteins in *Bmi1*^+/−^ mice but not in old WT mice. N = 2 independent experiments. (**h**) Quantification of the number of NeuN + cortical neurons in the frontal cortex of 15–20 month-old WT (n = 5) and *Bmi1*^+/−^ (n = 5) using IHC. (**i**) Quantification of the number of neurons positive for activated caspase-3 in the frontal cortex of 15–20 month-old WT (n = 5) and *Bmi1*^+/−^ (n = 5) mice. (**j**) Composite images of IHC staining on whole brain sections from 24-month old WT and *Bmi1*^+/−^ mice showing amyloid-positive deposits in the cortex (Ctx) and hippocampus (Hip) but not in the cerebellum (Cb) of *Bmi1*^+/−^ mice. (Right panel) High-resolution images showing extra-cellular amyloid plaque-like structures (arrow) in the cortex and hippocampus of *Bmi1*^+/−^ mice. Values are mean ± SEM. *P < 0.05; **P < 0.01; Student’s t-test.

We previously showed that most neuronal anomalies in *Bmi1*^−/−^ mice were mediated by p53 activation^18^. Using immuno-blot, we observed increased p53 levels in the cortex of 15–20 month-old *Bmi1*^+/−^ mice when compared to WT littermates (Fig. 3a). Hallmarks of AD and related dementias are accumulation of p-Tau, extra-cellular amyloid plaques, reduced synaptophysin immunoreactivity and neuronal loss. By immunoblot using antibodies that recognize p-Tau at Ser202 (AT-8) or Ser396 and Ser404 (PHF1), we observed the presence of p-Tau accumulation in *Bmi1*^+/−^ cortices (Fig. 3a). In cultured human neurons, *BMI1* knockdown results in an increase in total Tau levels^20^. Similarly, we observed that total Tau levels were increased in the cortex of 22–24 month-old *Bmi1*^+/−^ mice (Fig. S1a,b). Remarkably, these anomalies correlated with reduced synaptophysin levels (indicative of synaptic atrophy) and increased p-JNK immunoreactivity (Fig. 3a)^36,37^. By immuno-histochemistry (IHC), we observed p-Tau immunoreactivity in the neuron’s perikarya of 20 month-old WT and *Bmi1*^+/−^ mice. However, large p-Tau deposits in the neuronal cell body and strong immunoreactivity in track fibers of the cortical white matter were only observed in *Bmi1*^+/−^ mice (Fig. 3b). The presence of p-Tau tangles was not observed.

We next performed immunoblot on mouse cortices using an antibody (FCA3542) that can recognize the mouse and human amyloid peptide. Notably, we could detect the presence of a ~12–14 kDa band only in the *Bmi1*^+/−^ brain samples (Fig. 3c-Input 20%). This band was also revealed using FCA3542 after immunoprecipitation (IP) with another anti-amyloid antibody (DE2B4), but not after IP with IgG (Fig. 3c-IP). Antibody specificity was also confirmed using cortical extracts from human *APP*^*Swe/Dutch/Iowa*^ (*APP*) transgenic mice (Fig. 3c-IP). On *Bmi1*^+/−^ brain sections at 20 months of age, we observed that amyloid was preferentially found in the neuronal soma (Fig. 3d). Amyloid plaques were not observed. Cleavage of APP by the β-secretase Bace1 generates the ~12–14 kDa C99 fragment (C99). Notably, we observed increased Bace1 levels in cortical neurons of *Bmi1*^+/−^ mice (Fig. 3a,e). To distinguish between accelerated aging and neurodegeneration, we compared the brains of 24 month-old WT mice with that of 15 month-old *Bmi1*^+/−^ mice for the expression of the ~12–14 kDa fragment. Notably, the amyloid-related band was not detectable in the cortex of old WT mice (Fig. 3f). Likewise, p-JNK and p-Tau (AT-8) were not observed in the 24 month-old WT mice (Fig. 3g).

To evaluate the possibility of neuronal loss, we calculated the total number of cortical neurons per section in the frontal cortex using a pan-neuronal marker (NeuN). When compared to WT littermates, 15–20 month-old *Bmi1*^+/−^ mice displayed a ~20% reduction in NeuN-positive neurons (Fig. 3h). To examine if the lower neuronal density observed in *Bmi1*^+/−^ mice correlated with increased apoptosis, we quantified the number of neurons positive for activated caspase-3 in the frontal cortex. This revealed a ~2 fold increase in the frequency of apoptotic neurons in old *Bmi1*^+/−^ mice (Fig. 3i). Reduced neuronal density and increased apoptosis were also observed in the hippocampus of 15–17 month-old *Bmi1*^+/−^ mice (Fig. S2a,b), which was consistent with the behavioral and LTP results. We next analyzed the brains from rare surviving 22–25 month-old *Bmi1*^+/−^ mice using antibodies against amyloid (n = 7). A single 24 month-old *Bmi1*^+/−^ animal presented amyloid deposits that were present in all cortical areas, with other brain regions being generally less affected (Fig. 3j). Interestingly, the pattern of amyloid immuno-reactivity was slightly distinct when using another amyloid antibody (Fig. S3a,b). Intra-neuronal accumulation of amyloid and reduced synaptophysin immuno-reactivity was also observed in the hippocampus of another *Bmi1*^+/−^ mouse (Fig. S3c,d). This revealed that old *Bmi1*^+/−^ mice could spontaneously develop a disorder sharing some similarities with AD.

### Bmi1 hemi-deficiency cooperates with APP-mediated neurodegeneration

**Figure 4:**
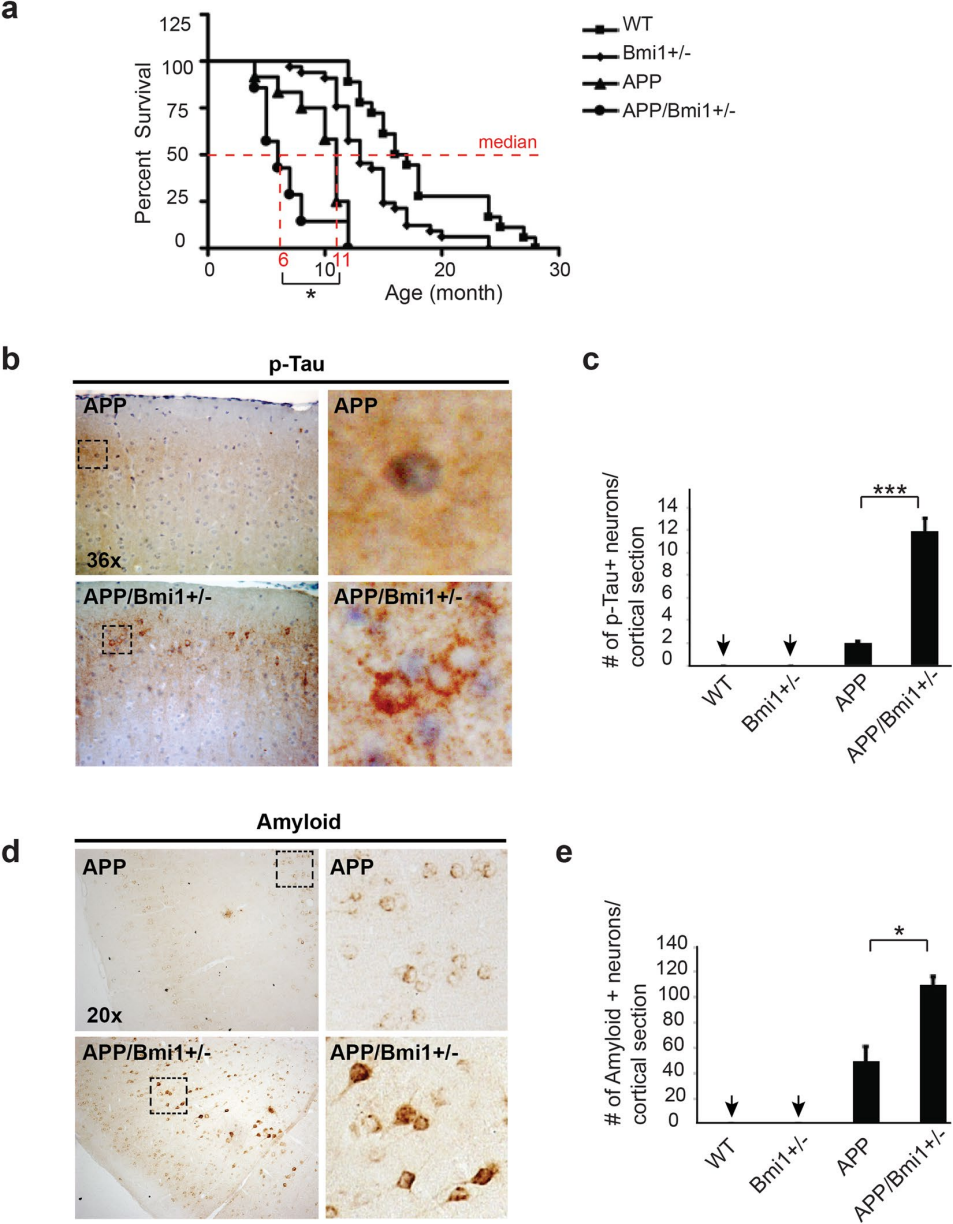
*APP/Bmi1*^+/−^ mice show genetic cooperation in disease onset and severity. (**a**) Kaplan-Meyer representation of the survival curves of WT (n = 11), *Bmi1*^+/−^ (n = 13), *APP* (n = 6) and *APP/Bmi1*^+/−^ (n = 6) mice. (**b**) IHC staining for p-Tau (PHF1) on cortical sections of 6 month-old WT (n = 3), *Bmi1*^+/−^ (n = 3), *APP* (n = 3),and *APP/Bmi1*^+/−^ (n = 3) mice. Scale bar: 20μm. Scale bar in the inset: 8μm. (**c**) Quantification of p-Tau-positive neurons shown in (**b**). (**d**) IHC for amyloid (DE2B4) on cortical sections of 6-month old WT (n = 3), *Bmi1*^+/−^ (n = 3), *APP* (n = 3), and *APP/Bmi1*^+/−^ (n = 3) mice. Scale bar: 20μm. (**e**) Quantification of amyloid-positive cells shown in (**d**). Values are mean ± SEM. *P < 0.05; (**) < 0.01; (***) < 0.001; Student’s t-test.

Genetic cooperation between two mutations can be indicative of protein-protein interactions and/or convergent molecular pathways. To test if *Bmi1* hemi-deficiency could cooperate or not with FAD-associated mutations, we crossed *Bmi1*^+/−^ mice with human *APP* transgenic mice. From this, we obtained 4 genotypes in F1: WT, *Bmi1*^+/−^, *APP* and *APP/Bmi1*^+/−^ mice. Kaplan Mayer analysis revealed that although both *Bmi1*^+/−^ and *APP* mice had reduced maximal and median lifespan compared to WT littermates, the median lifespan of the *APP/Bmi1*^+/−^ animals was further reduced by ~45% when compared to *APP* mice (Fig. 4a). The difference was significant despite the limitations of using a small data set. When analyzed by IHC at 6 months of age for p-Tau, we observed almost no signal in WT and *Bmi1*^+/−^ mice, and weak immunoreactivity in *APP* mice. Notably, while neuronal accumulation of p-Tau was rare in *APP* mice and absent in *Bmi1*^+/−^ mice, it was relatively robust and abundant in *APP/Bmi1*^+/−^ mice (Fig. 4b,c). Similarly, although intra-neuronal amyloid immunoreactivity was weak to absent in *Bmi1*^+/−^ mice at this young age, it was easily detectable in *APP* mice (Fig. 4d). Notably, the number of immunoreactive neurons and the signal strength for amyloid were greatly enhanced in *APP/Bmi1*^+/−^ mice (Fig. 4d,e). Other parameters of disease severity were also enhanced in *APP/Bmi1*^+/−^ mice, including reactive gliosis, microglia activation, synaptic atrophy and apoptosis (Fig. S4). Taken together, these results suggested genetic cooperation between *Bmi1* hemi-deficiency and mutant *APP* in neurodegeneration.

### Loss of heterochromatin and DDR in cortical neurons of *Bmi1*^+/−^ mice

**Figure 5:**
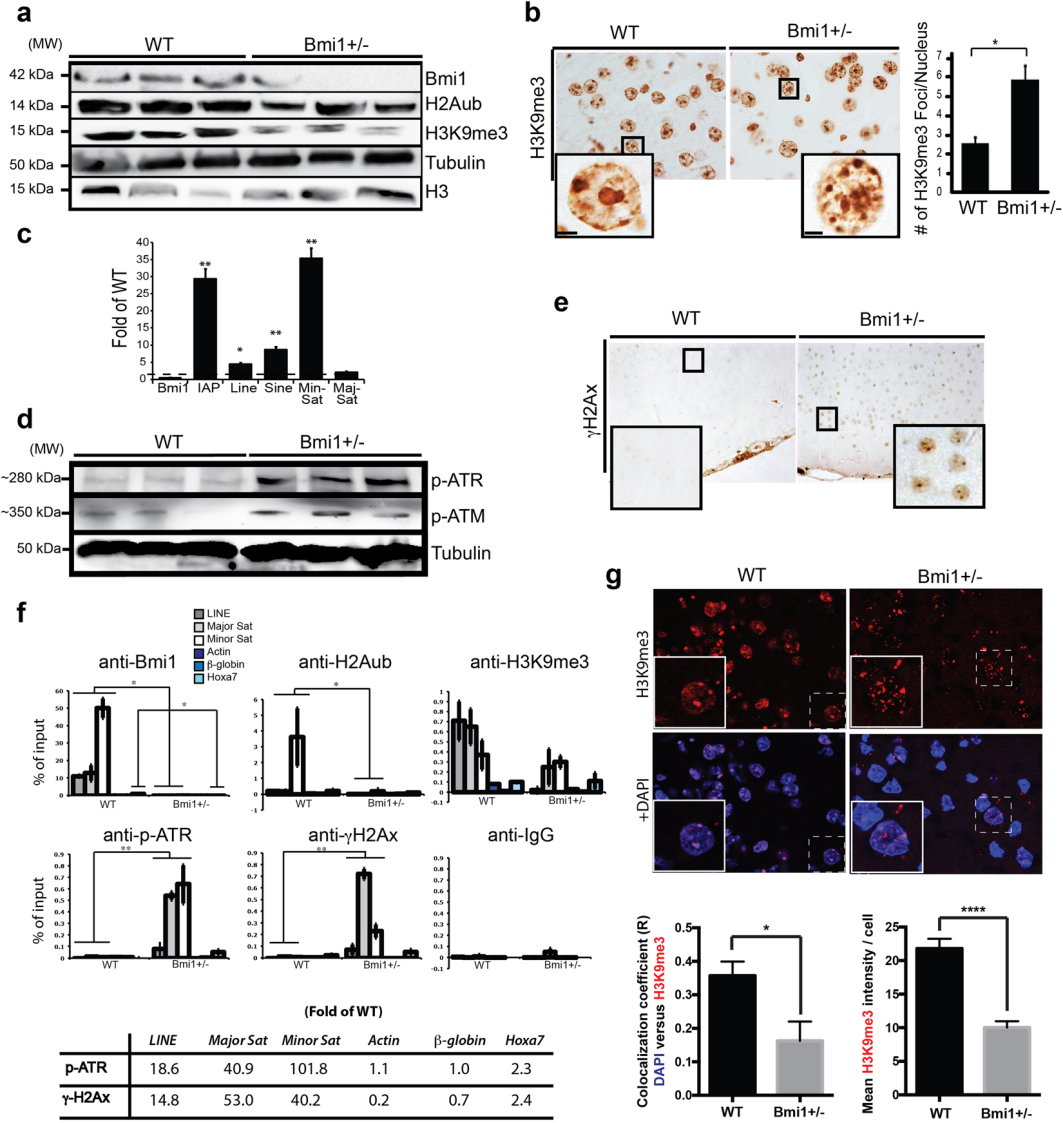
Loss of heterochromatin and DDR in cortical neurons of *Bmi1*^+/−^ mice. (**a**) Western blot analysis of cortical extracts from old WT (n = 6) and *Bmi1*^+/−^ (n = 6) mice. (**b**) IHC on cortical sections from old WT (n = 3) and *Bmi1*^+/−^ (n = 3) mice with quantification of the number of H3K9^me3^ foci per nucleus (6 sections/sample were counted). Scale bar in the inset: 3μm. (**c**) Real-time RT-PCR analysis for the expression of satellite repeats in cortices from WT (n = 3) and *Bmi1*^+/−^ (n = 3) mice. (**d**) Western blot analysis of cortical extracts from old WT (n = 3) and *Bmi1*^+/−^ (n = 3) mice. (**e**) IHC for γH2AX on cortical sections of old WT and *Bmi1*^+/−^ mice. (**f**) ChIP analyses were performed on frontal cortex homogenates from 15 month-old WT (n = 3) and *Bmi1*^+/−^ (n = 3) mice. Quantitative PCR was performed in triplicate for each DNA sequence. All data are presented as fold of input. Bmi1 and H2A^ub^ reduction (p < 0.05), and p-ATR and γH2AX accumulation (p < 0.01) in *Bmi1*^+/−^ mice are significant (Two-way ANOVA analysis). (bottom panel) Fold differences for γH2AX and p-ATR accumulation in *Bmi1*^+/−^ mice were measured relative to WT levels for each DNA sequences. (**g**) Cortical slices from 45-day old mice. Areas showed are located in layers 2-3 of the frontal cortex. In WT samples, large pyramidal neurons were strongly labeled by the H3K9^me3^ antibody and H3K9^me3^ co-localized with DAPI. In *Bmi1*^+/−^ samples, large pyramidal neurons were weakly labeled by the H3K9^me3^ antibody and the H3K9me3 signal was fragmented. Bottom panels: Quantification of the images showed in (**f**). Values are mean ± SEM. *P < 0.05; **P < 0.01; ****P < 0.0001; Student’s t-test.

BMI1 is required for heterochromatin maintenance in somatic cells^19^. We thus investigated if the AD-like phenotype of *Bmi1*^+/−^ mice was also associated with heterochromatin alterations. By Western blot analysis on whole cortices, we observed reduced levels for histones H2A^ub^ and H3K9^me3^ in 15 month-old *Bmi1*^+/−^ mice (Fig. 5a). To test if this correlated with an abnormal chromatin structure, we performed IHC. We observed that in contrast to WT mice, where 2-3 large chromocenters/neuron were present, chromocenters of *Bmi1*^+/−^ neurons were smaller and more numerous, suggesting heterochromatin de-nucleation (Fig. 5b). Consistently, this was associated with transcriptional de-repression of satellite repeats, which are normally silenced by heterochromatin (Fig. 5c)^38–40^. One prediction of heterochromatin de-nucleation is activation of a DDR. The *Ataxia Telengectasia Mutated* (ATM) and *Ataxia Telengectasia and RAD-3 Related* (ATR) kinases work at the apex of the DDR^41–43^. Using immunoblot, we observed accumulation of phospho-ATM at Serine 1981 (p-ATM) and phospho-ATR at Serine 428 (p-ATR) in the cortices of *Bmi1*^+/−^ mice (Fig. 5d). The histone mark γH2AX is present at sites of DNA damage^44^. We observed nuclear accumulation of γH2AX in cortical neurons only in *Bmi1*^+/−^ mice, suggesting that the DDR occurs primarily in neurons (Fig. 5e).

To localize DNA damage on the genome, we performed chromatin immuno-precipitation (ChIP) experiments on whole cortices. As expected, Bmi1, H3K9^me3^ and H2A^ub^ were enriched at genomic repeats (Minor and Major satellite repeats and LINE elements) and at the *Hoxa7* locus in WT mice and were depleted in *Bmi1*^+/−^ mice (Fig. 5f)^19^. In contrast, while p-ATR and γH2AX were nearly absent in WT mice, they were preferentially and highly enriched at genomic repeats in *Bmi1*^+/−^ mice (Fig. 5f).

To test if heterochromatin anomalies were present before disease onset, we performed immuno-blot, IHC and confocal microscopy on cortices from 2-3 month-old WT and *Bmi1*^+/−^ mice. This revealed reduced H3K9^me3^ levels and fragmented H3K9^me3^ immunostaining in *Bmi1*^+/−^ pyramidal neurons when compared to WT (Figs 5g and S5a,b). Reduced H3K9^me3^ levels were also present in cultured embryonic day 18.5 neurons, as measured using ChIP analyses (Fig. S5c). Despite the presence of chromatin anomalies at the earliest step of neurogenesis, we failed to detect a DDR or the expression of AD-related markers in 3 month-old *Bmi1*^+/−^ mice using immuno-blot (Fig. S5d). Mild accumulation of p53 was however noticed in *Bmi1*^+/−^ mice (Fig. S5d). This suggested that in *Bmi1*^+/−^ mice, loss of heterochromatin is a very early event in the disease process.

### Amyloid and Tau pathologies can be mitigate by inhibition of the DDR

**Figure 6:**
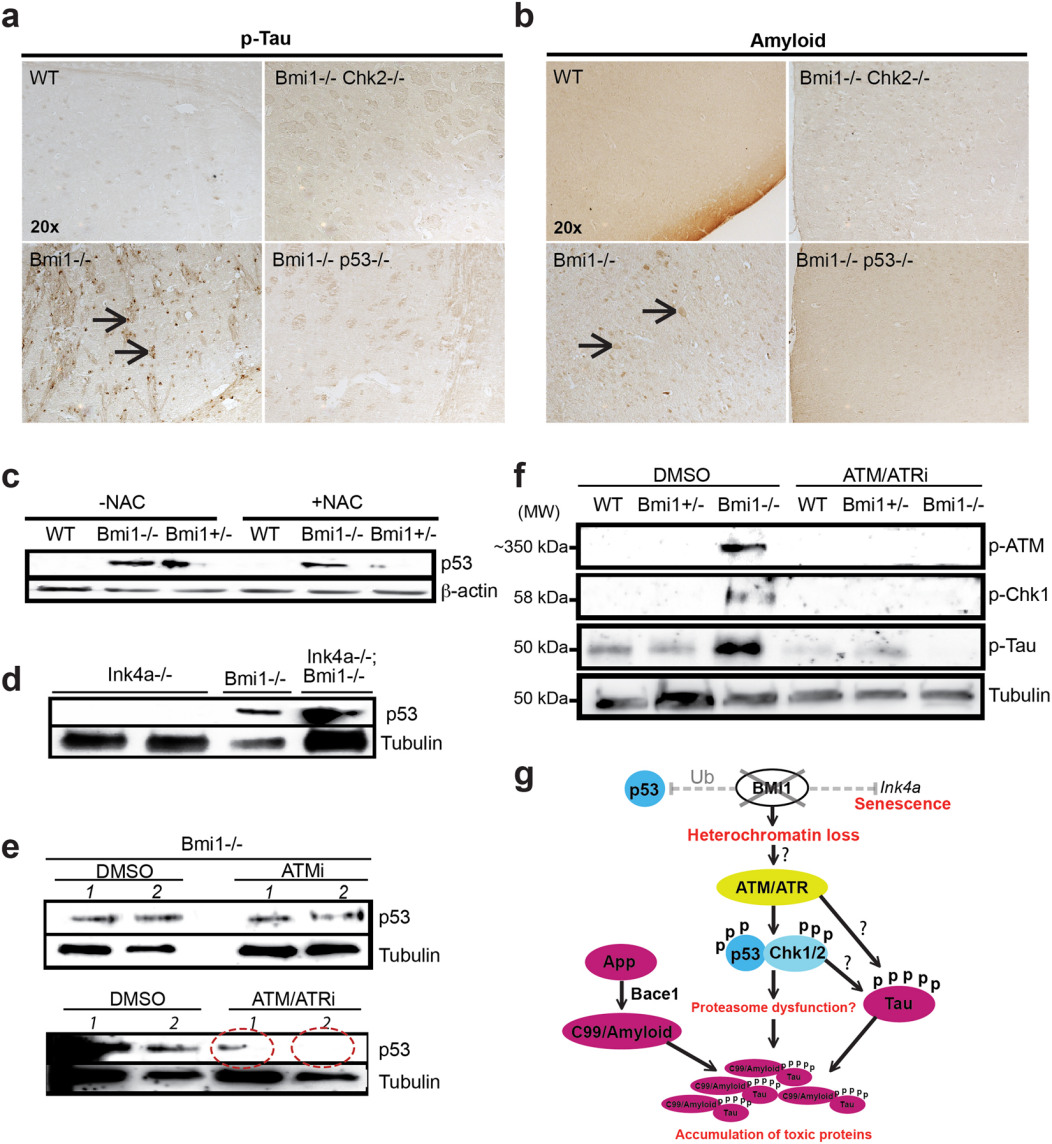
Blocking the DDR mitigate the amyloid and Tau phenotype in *Bmi1*-null neurons. (**a**,**b**) IHC performed on cortical sections from 25 day-old mice. (**a**) Arrows indicate p-Tau-positive neurons in *Bmi1*^−/−^ mice. (**b**) Arrows indicate amyloid-positive neurons in *Bmi1*^−/−^ mice. These anomalies were largely prevented in the double-mutant mice. (**c**–**f**) Immunoblot were performed on embryonic day 18.5 mouse cortical neurons cultured for 7 days *in vitro*. (**c**–**d**) Addition of NAC or co-deletion of *Ink4a* could not prevent p53 accumulation in *Bmi1*-deficient neurons. Note the presence of p53 also in *Bmi1*^+/−^ neurons. NAC was added daily to the cultures for a period of 7 days. (**e**,**f**) DMSO, ATMi or ATM/ATRi were added to the cultures 16 hours prior to protein extraction. (**e**) ATM/ATRi largely prevented p53 accumulation in *Bmi1*^−/−^ neurons. Numbers *1* and *2* in (**e**) indicate two independent biological replicates. Note residual p53 expression in ATM/ATRi-treated neurons (circles with dash lines). (**f**) Addition of ATM/ATRi could prevent p-ATM, p-Chk1 and p-Tau accumulation in *Bmi1*^−/−^ neurons. (**g**) Hypothetical model to explain the impact of *Bmi1* inactivation in mouse neurons. Following loss of *Bmi1*, up-regulation of the *Ink4a* locus induces neuronal senescence. On the other hand, genomic instability at the heterochromatin induces a chronic DDR. The p53 protein is then stabilized by ATR/ATM-mediated kinase activity. The accumulation of p53 may be also exacerbated by the loss of Bmi1/Ring1a-mediated ubiquitin ligase and antioxidant activities. The activated ATM/Chk2, ATR/Chk1 and p53 pathways then promote Tau phosphorylation and accumulation of the C99 fragment. Elevated Bace1 levels also contribute to over-production of the C99 fragment. Accumulation of these toxic proteins contributes to neurodegeneration.

*Bmi1*-null (*Bmi1*^−/−^*)* mice are smaller than normal and die around post-natal day 30^12^. Yet, we could find cortical neurons immuno-reactive for p-Tau and amyloid in 25 day-old *Bmi1*^−/−^ mice (Fig. 6a,b). To gain insight into the disease mechanism, we generated cohorts of 25 day-old *Bmi1*^−/−^ mice carrying null mutations in *p53* (*Bmi1*^−/−^*/p53*^−/−^*)* or *Chk2* (*Bmi1*^−/−^*/Chk2*^−/−^). *Chk2* encodes a kinase activated by ATM, and both ATM and Chk2 can stimulate p53 activity^45,46^. Notably, we found that p-Tau and amyloid immuno-reactivity were both mitigated in *Bmi1*^−/−^*/p53*^−/−^and in *Bmi1*^−/−^*/Chk2*^−/−^ mice (Fig. 6a,b).

To understand the basis of p53 activation, we cultured WT, *Bmi1*^−/−^ and *Bmi1*^+/−^ embryonic day 18.5 cortical neurons to test if free radical-mediated DNA damage was responsible for p53 accumulation^18,47,48^. Using N-Acetyl Cysteine (NAC), a free radical scavenger, we found that reducing free radicals could slightly improve p53 levels in *Bmi1*^−/−^ and *Bmi1*^+/−^ neurons (Fig. 6c). The p19^Arf^ protein can stabilize p53, and p19^Arf^ is up regulated in *Bmi1*^−/−^ neurons^18,49^. However, co-deletion of *Bmi1* and *Ink4a* (encoding for p16^Ink4a^ and p19^Arf^) could not prevent p53 accumulation (Fig. 6d). In response to DNA damage, the ATM and ATR kinases can target p53 for phosphorylation^45,50,51^. We found that inhibition of ATM alone could not prevent p53 accumulation, but that inhibition of ATM and ATR largely mitigated p53 accumulation in *Bmi1*^−/−^ neurons (Fig. 6e). Residual p53 levels were however observed. We also observed the presence of p-ATM and phospho-Chk1 (a downstream target of ATR) in *Bmi1*^−/−^ neurons and found that ATM/ATRi was sufficient to prevent their accumulation (Fig. 6f). Remarkably, p-Tau accumulation in *Bmi1*^−/−^ neurons could be also prevented after exposure to ATM/ATRi (Fig. 6f). These results suggested a theoretical model where AD-related neuronal pathologies observed in *Bmi1*-deficient mice are triggered in part by a ATM/ATR-driven DDR working upstream of p53, but where Bmi1/Ring1 ubiquitin ligase activity and Bmi1-mediated ROS repression are also likely required to prevent p53 accumulation (Fig. 6g)^52^.

### Loss of heterochromatin and DDR in AD brains

**Figure 7:**
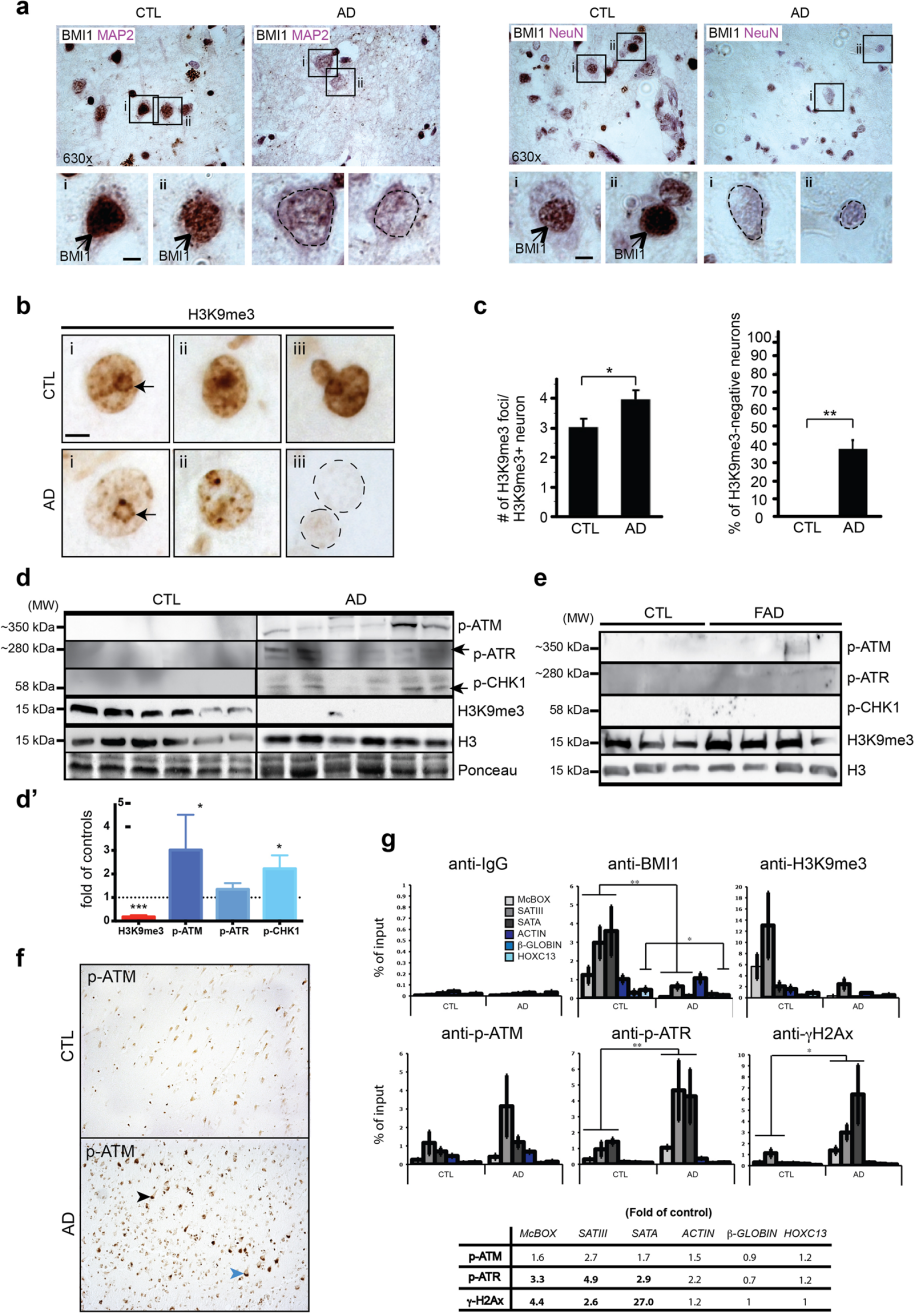
Loss of heterochromatin and DDR in AD brains. (**a**) IHC staining for BMI1 (brown staining-black arrows) and MAP2 or NeuN (violet staining) on paraffin sections from the frontal cortex of age-match control (n = 2) and AD (n = 2) patients. Weak nuclear staining for BMI1 (dash lines) was observed in AD neurons. Scale bar: 3μm. (**b**) IHC staining for H3K9^me3^ on frontal cortex sections of control (n = 5) and AD (n = 5) brains: (i) reduced peri-nucleolar heterochromatin in AD (arrows), (ii) chromocenters de-condensation in AD, and (iii) loss of H3K9^me3^ immunoreactivity in AD neurons (dash lines). Scale bar: 5μm. (**c**) Quantification of data in (b) showing the number of H3K9^me3^ foci/H3K9^me3^-positive neuron. H3K9^me3^-negative neurons were excluded from the analysis. Quantification of the number of H3K9^me3^-negative neurons, which were not found in aged controls. 6 full-fields at 630 magnification/sample were counted. Values are mean ± SEM. *P < 0.05; (**) < 0.01; Student’s t-test. (**d**) Immunoblot using extracts from the frontal cortex of age-match control and AD patients. (**d’**) Quantification of the results showed in (**d**). (**e**) Immunoblot using extracts from the hippocampus of age-match control and FAD patients. (**f**) IHC staining for p-ATM on paraffin sections from the frontal cortex of age-match control (n = 2) and AD (n = 2) patients. p-ATM is predominant in AD neurons and accumulates in both cytosolic (blue arrowhead) and nuclear (black arrowhead) compartments. (**g**) ChIP experiments were performed on frontal cortex extracts of age-match control (n = 6) and AD brains (n = 6). All data are represented as *fold of input*. (A) Note BMI1 and H3K9^me3^ enrichment at *McBOX*, *SATIII* and *SATA* in control samples. BMI1 was also found at *HOXC13*. BMI1 and H3K9^me3^ were depleted at all loci in AD samples (bottom). Fold differences between control and AD samples for p-ATM, p-ATR and γH2AX accumulation. Values are mean ± SEM. *P < 0.05; **P < 0.01; ***P < 0.001; Two way-ANOVA test was performed for multiple gene analysis and Student’s t-test for single gene analysis.

We next investigated the possible relevance of our findings in the context of AD. We first confirmed the robust expression of BMI1 in cortical neurons from aged controls and BMI1 depletion in neurons from AD patients (Fig. 7a). To test for possible heterochromatin anomalies, we performed IHC on frontal cortex sections^53^. In control samples, all neurons were labeled with H3K9^me3^ and presented ~3 heterochromatic foci (or chromocenters)/neuron (Fig. 7b,c). In AD samples, ~38% of all pyramidal neurons showed highly reduced or absent H3K9^me3^ nuclear staining, and the neuron’s chromocenters were smaller and more numerous (Fig. 7b,c). To test the possibility of genomic instability, we analyzed the expression of DDR proteins. By Western blot analysis, we observed accumulation of p-ATM, p-ATR and p-CHK1 in AD brains, but not in age-match controls (Fig. 7d,d’). The presence of a DDR in AD brains also correlated with reduced H3K9^me3^ levels (Fig. 7d). In contrast, accumulation of DDR proteins was not observed in FAD brains, with the exception of one sample presenting p-ATM accumulation (Fig. 7e). Likewise, H3K9^me3^ levels were unaffected in FAD brains (Fig. 7e). Using IHC, we also confirmed that DDR in AD brains occurred primarily in neurons (Fig. 7f).

To establish where the DDR was located on the genome, we performed ChIP experiments using frontal cortex samples. When compared to controls, we found significant enrichment for p-ATR and γH2AX at genomic repeats (*SATIII*, *SATA* and *McBOX*) in AD brains (Fig. 7g). In contrast, there was no enrichment at *HOXC13*, β*-GLOBIN* or *ACTIN* (Fig. 7g). Although there was a general trend for p-ATM enrichment at genomic repeats in AD samples, it was not significant. BMI1 and H3K9^me3^ were also enriched at *HOXC13* and at genomic repeats in control brains and were depleted in AD brains (Fig. 7g). Taken together, these findings revealed remarkable similarities between the neuronal genomic instability phenotype of *Bmi1*^+/−^ mice and AD patients.

## Discussion

We have found that *Bmi1*^+/−^ mice develop with age a neurological and progeroid syndrome characterized by alopecia, weight loss, abnormal paw clasping reflex and neuronal senescence. Memory deficit as measured by their performance in the Morris water maze and Barnes test correlated with impairment in LTP formation as well as with synaptic atrophy and neuronal loss. Pathological brain anomalies such as p-Tau and amyloid accumulation were also found in old *Bmi1*^+/−^ mice. Remarkably, when compared to *Bmi1*^+/−^ or *APP* mice alone, *Bmi1*^+/−^/*APP* mice showed significantly shorter lifespan and increased disease severity. Genetic and cell culture studies further revealed that inhibition of the DDR and/or p53 could mitigate the accumulation of p-Tau and amyloid in neurons from *Bmi1*-null mice, suggesting that the neuronal response to genomic instability plays a role in the disease process. At last, loss of heterochromatin in neurons and accumulation of DNA damage at genomic repeats were found in *Bmi1*^*+/−*^ mice and AD brains.

One possible interpretation of our data is that *Bmi1*^+/−^ mice age faster and thus present age-related neurological anomalies earlier than WT mice. This possibility is supported by our observation that some pathological marks, including p-Tau (using PHF1, which recognizes an epitope around Ser396 and Ser404) and SA β-galactosidase activity, although much less abundant, were also detected in the brains of old WT mice. However, other pathological marks, such as p-Tau (using AT-8, which recognizes Ser202), p-JNK and amyloid (C99) accumulation were not detected even in 24 month-old WT mice, suggesting that *Bmi1* hemi-deficiency represents a pathological brain aging process that is distinct from end-stage physiological brain aging.

Using the *Bmi1*^+/−^ and *Bmi1*^−/−^ mouse models, we observed activation of DDR proteins and of p53 in neurons. This is consistent with previous work showing that *Bmi*1 is important to maintain genomic stability^15–18,54^. Interestingly, over-expression of *Chk1* or *Chk*2 was shown to exacerbate Tau toxicity in a *Drosophila* model of neurodegeneration by specific phosphorylation of human Tau at Ser262^55^. Likewise, JNK activation may promote Tau phosphorylation^32^. Thus, several kinases potentially targeting Tau are activated in *Bmi1*^+/−^ mice. It however remains to be investigated by which mechanisms Bace1 level is increased and why the amyloid metabolism is affected in *Bmi1*^+/−^ mice. Our results showed that amyloid accumulation was mitigated after *p53* deletion and we recently reported that p53 activation in human neurons might perturb the clearance of misfolded proteins by interfering with proteasome function^20^. Thus, a plausible mechanism to explain our results is that activated p53 also impairs proteasome function in mouse neurons. Further work is however needed to validate this.

The heterochromatin island hypothesis of aging stipulates that epigenome instability at the constitutive heterochromatin is a driving force of cellular aging^56,57^. This hypothesis may be particularly relevant in post-mitotic neurons, where cell division associated with telomere attrition is irrelevant to the cellular aging process. Notably, inactivation of chromatin remodeling proteins, such as those of the NURD complex, results in DDR and in cellular senescence that are preceded by heterochromatin alterations^58^. Furthermore, modifications of constitutive heterochromatin in response to an acute stress can result in epigenome instability and perturbation of gene expression program, which are hallmarks of cellular aging^59^. In mice, deletion of the SUV39h1/h2 methyltransferases leads to reduced viability, loss of H3K9^me3^ and genomic instability^60^. In *Drosophila*, perturbation of H3K9^me2^ levels through inactivation of Su(var)3-9 results in genomic instability and constitutive DDR at the heterochromatin, in both somatic and germ-line cells^61^. We showed here that heterochromatin alterations in *Bmi1*^+/−^ mice are present at the time of neurogenesis and thus before activation of DDR proteins and neurodegeneration. Notably, this correlated with preferential accumulation of DNA damage (as marked by γH2Ax) at repetitive DNA sequences. Similarly, we found that DNA damage accumulation was predominant at genomic repeats in AD brains. Exactly why loss of heterochromatin compaction strongly correlates with DNA damage accumulation at genomic repeats is unknown. It is possible that repetitive DNA sequences are intrinsically unstable upon loss of heterochromatin compaction and thus prone to *de novo* DNA damage formation. It is also possible that the efficiency of DNA repair is reduced upon BMI1 deficiency or that recognition of the H3K9^me3^ mark is important to activate the process of DNA repair at these specific regions^15,16,62^. It remains however to be tested whether heterochromatin anomalies observed in *Bmi1*^+/−^ mice are sufficient to induce the DDR and neurodegeneration.

Interstitial amyloid plaques and Tau tangles represent the classical pathological hallmarks of AD. While synaptic atrophy, neuronal loss and behavioral anomalies are present in 15-month old *Bmi1*^+/−^ mice, the animals do not develop Tau tangles or amyloid plaques (with the exception of one animal). Rather, old *Bmi1*^+/−^ mice present intra-neuronal accumulation of amyloid and show moderate levels of p-Tau deposits in the neuronal cell body and the axon. The absence of amyloid plaques formation in *Bmi1*^+/−^ mice is not surprising since the mouse amyloid peptide is known to be less prone to aggregation than the human one^63^. Also, transgenic mice over-expressing wild-type human *APP* rarely develop amyloid plaques, in contrast with those over-expressing *APP* with FAD-associated mutations^64^. Notably, massive neurodegeneration was reported in *APP/PSEN1* transgenic mice (*APP(SL)PS1KI* mice), which only develop intra-neuronal accumulation of amyloid^65^. Thus, intra-neuronal accumulation of oligomeric amyloid species, including the C99 fragment, may be sufficient to induce neurodegeneration^66,67^. Nevertheless, the absence of amyloid plaques and Tau tangles as well as the late onset neuronal disease should be taken into account before using *Bmi1*^+/−^ mice as a model of AD.

In conclusion, we demonstrated that loss of one *Bmi1* allele in mice results in age-related neurodegeneration sharing some similarities with AD. *Bmi1*^+/−^ mice may thus represent an interesting animal model to identify new pathogenic mechanisms related to AD.

## Supplementary information

Supplementary Information

## Data Availability

The authors declare that they will make available all data presented in this manuscript.
