# Supplementary material for: Heterochromatic genome instability and neurodegeneration sharing similarities with Alzheimer’s disease in old Bmi1+/− mice
Source: Sci Rep. 2019 Jan 24;9:594. doi: 10.1038/s41598-018-37444-3 (PMC6346086; doi:10.1038/s41598-018-37444-3)
Supplement: Supplementary file 1 — Supplementary Information [file 41598_2018_37444_MOESM1_ESM.pdf]

Heterochromatic genome instability and neurodegeneration sharing similarities with Alzheimer's disease in old *Bmi1*+/- mice

Jida El Hajjar[1], Wassim Chatoo[1], Roy Hanna[1], Patrick Nkanza[1], Nicolas Tétreault[1], Yiu Chung Tse[2,3], Tak Pan Wong[2,3], Mohamed Abdouh[1], and Gilbert Bernier[1,4,*]

[1] Stem Cell and Developmental Biology Laboratory, Maisonneuve-Rosemont Hospital, 5415 Boul. l'Assomption, Montreal, Canada, H1T 2M4

[2] Department of Psychiatry, McGill University, Montreal, Canada

[3] Douglas Mental Health University Institute, Montreal, Canada

[4] Department of Neurosciences, University of Montreal, Montreal, Canada

*Corresponding author:

Tel: 514-252-3400 ext. 4648

Fax: 514-253-7626

E-mail: gbernier.hmr@ssss.gouv.qc.ca

Content:

Supplementary Figures Legends

5 Supplementary Figures

1 Supplementary Table

SUPPLEMENTAL FIGURE LEGENDS

Figure S1. Old *Bmi1*$^{+/-}$ mice show increased total Tau levels in the cortex

(a) Western blot analysis of cortices from 22-27 month (M)-old WT and *Bmi1*$^{+/-}$ mice revealing elevated Tau levels (k9JA antibody).

(b) Quantification of results presented in (a). The Bmi1/GAPDH and Tau/GAPDH ratio were normalized to 1 for WT mice, revealing the relative expression levels in *Bmi1*$^{+/-}$ mice. Values are mean ± SEM. (*) $P<0.05$; (**)<0.01; Student's t-test.

Figure S2. Old *Bmi1*$^{+/-}$ mice show reduced number of neurons in the hippocampus

(a) IHC staining for Bmi1 in the hippocampus (CA2 and dentate gyrus (DG)). Note the reduced Bmi1 expression level and reduced number of neurons in the *Bmi1*$^{+/-}$ (17 month-old) mouse when compared to the WT (26 month-old).

(b) Quantification of the results showed in (a). Results are the sum of quantification of 3 slides/mouse, for a total of 2 *Bmi1*$^{+/-}$ mice (16 and 17 month-old) and 2 WT mice (24 and 26 month-old).

(c) Quantification of the number of neurons positive for activated caspase-3 (Casp3). Results are the sum of quantification of 3 slides/mouse, for a total of 2 *Bmi1*$^{+/-}$ mice (16 and 17 month-old) and 2 WT mice (24 and 26 month-old). Values are mean ± SEM. (*) $P<0.05$; (**)<0.01; Student's t-test.

Figure S3. Some very old *Bmi1*$^{+/-}$ mice present extra-cellular amyloid accumulations

(a-b) Note the accumulation of amyloid deposits in the cortex and hippocampus of an old *Bmi1*$^{+/-}$ mouse (24 months) as shown using the DE2B4 antibody.

(b) High-resolution composite (top) and high-resolution cropped (bottom) images from (a) showing extra-cellular amyloid plaque-like accumulations (black arrows) in the hippocampus of an old *Bmi1*$^{+/-}$ mouse.

(c) Intra-cellular accumulation of amyloid (MOAB2 antibody) in another 24 month-old *Bmi1*$^{+/-}$ mouse (arrows).

(d) Composite image showing reduced synaptophysin immuno-reactivity in the hippocampus of an old *Bmi1*$^{+/-}$ mouse (same animals as in (c)).

Figure S4. Reactive gliosis and microglia activation in *APP/Bmi1*$^{+/-}$ mice

IHC staining for GFAP (astrocytes), Iba1 (microglia), activated caspase-3 (apoptosis) and synaptophysin (synapses) on cortical sections of 6 month-old WT, *Bmi1*$^{+/-}$, *APP*, and *APP*/*Bmi1*$^{+/-}$ mice. Note the increase in staining intensity and in the number of positive cells for GFAP, Iba1 and activated caspase-3 in *APP*/*Bmi1*$^{+/-}$ mice when compared to the 3 other genotypes (arrows). In contrast, note the reduced immunoreactivity for synaptophysin in *APP*/*Bmi1*$^{+/-}$ mice when compared to the 3 other genotypes.

Figure S5. Loss of heterochromatin in *Bmi1*$^{+/-}$ neurons is an early event in the disease process

(a) Western blot on cortical extracts of 3 month-old WT and *Bmi1*$^{+/-}$ with quantification of results obtained (left).

(b) IHC on cortical sections from 3 month-old WT and *Bmi1*$^{+/-}$ showing loss of heterochromatin compaction in *Bmi1*$^{+/-}$ mice, with quantification of the results obtained shown at the left. Scale bar: 5µm. Values are mean ± SEM. (*) $P<0.05$; (**)<0.01; Student's t-test.

(c) ChIP analysis on 18.5 embryonic day WT (n = 3) and *Bmi1*$^{+/-}$ (n = 3) cortical neurons. Quantitative PCR was performed in triplicate for each DNA sequence. All data are presented as fold of input. Two-way ANOVA analysis exposed a significant decrease of Bmi1, H2A^{ub} and H3K9^{me3} accumulation at genomic repeats in *Bmi1*$^{+/-}$ neurons. (*) $P<0.01$.

(d) Western blot analysis of cortices from 3 month-old WT and *Bmi1*$^{+/-}$ mice showing undetectable expression of AD-related pathological markers in *Bmi1*$^{+/-}$ mice. Very faint p53 expression is however possibly present in the *Bmi1*$^{+/-}$ samples. Cortices from 15 month-old *Bmi1*$^{+/-}$ mice were used as positive control.

a

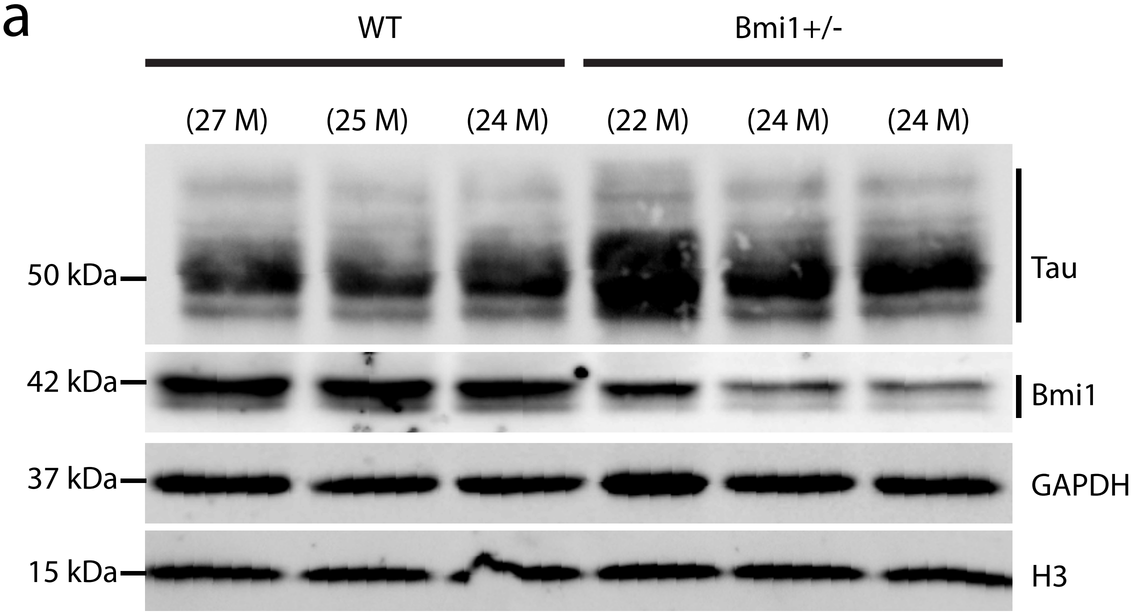
WT
Bmi1+/-
(27 M)
(25 M)
(24 M)
(22 M)
(24 M)
(24 M)
50 kDa
42 kDa
37 kDa
15 kDa
Tau
Bmi1
GAPDH
H3

b

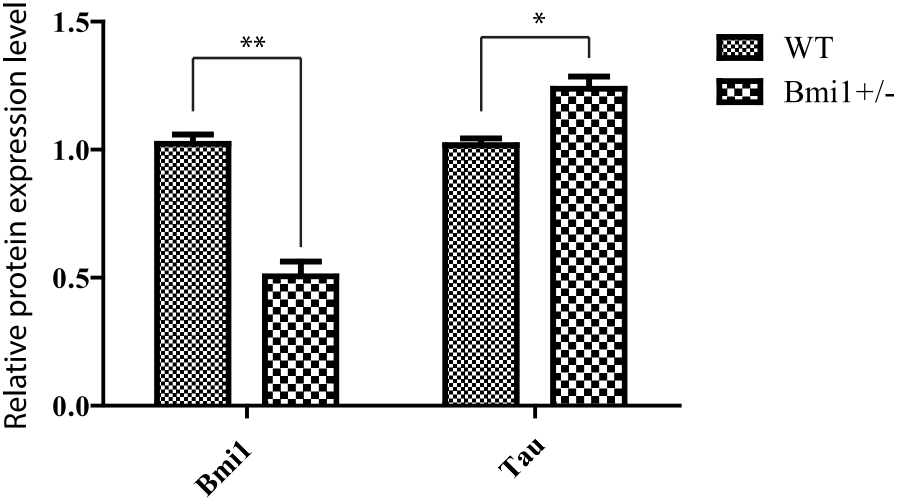
Relative protein expression level
1.5
1.0
0.5
0.0
**
*
Bmi1
Tau
WT
Bmi1+/-

Figure S1

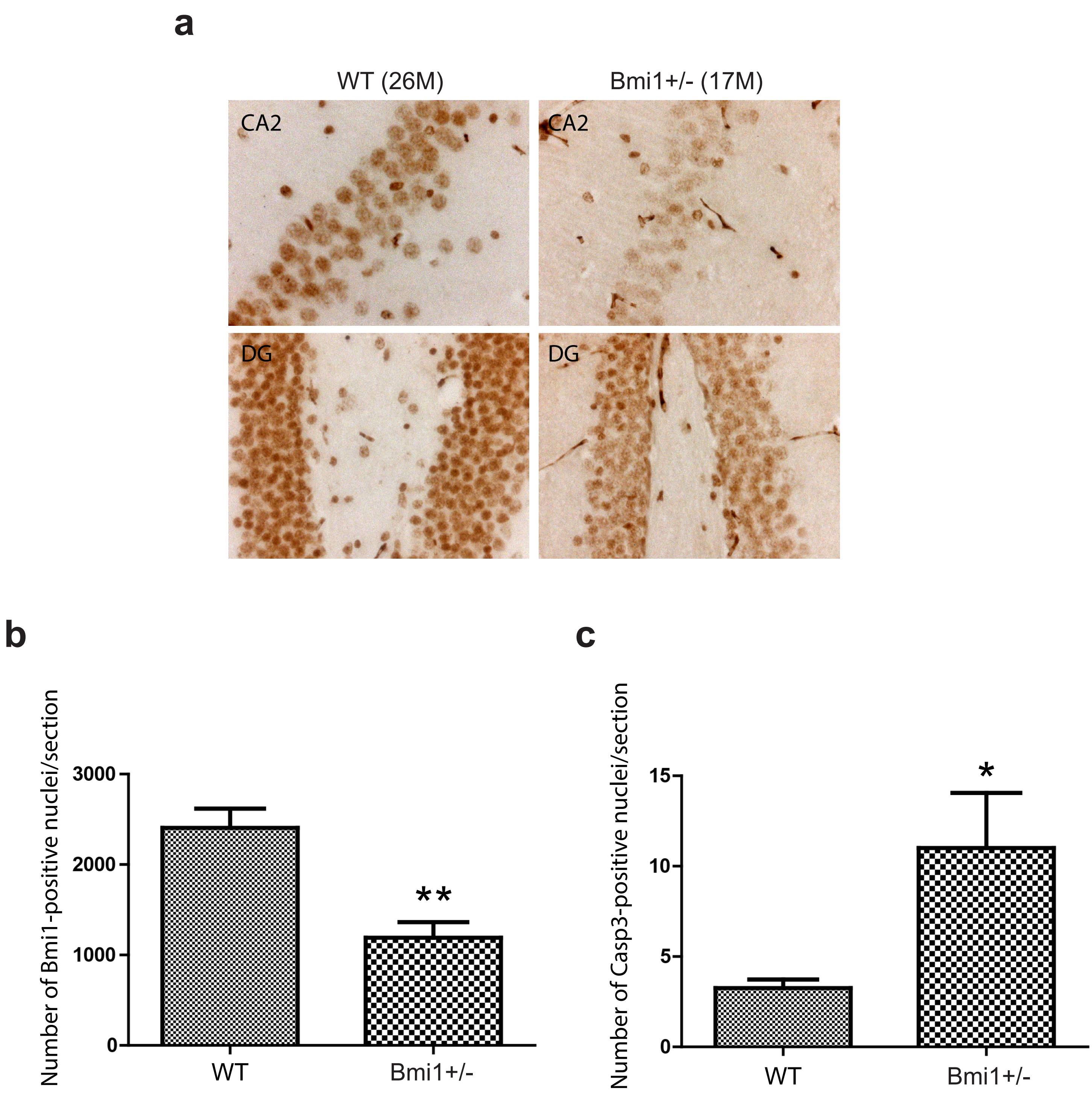
a
WT (26M)
Bmi1+/- (17M)
CA2
CA2
DG
DG
b
Number of Bmi1-positive nuclei/section
3000
2000
1000
0
**
WT
Bmi1+/-
c
Number of Casp3-positive nuclei/section
15
10
5
0
*
WT
Bmi1+/-

Figure S2

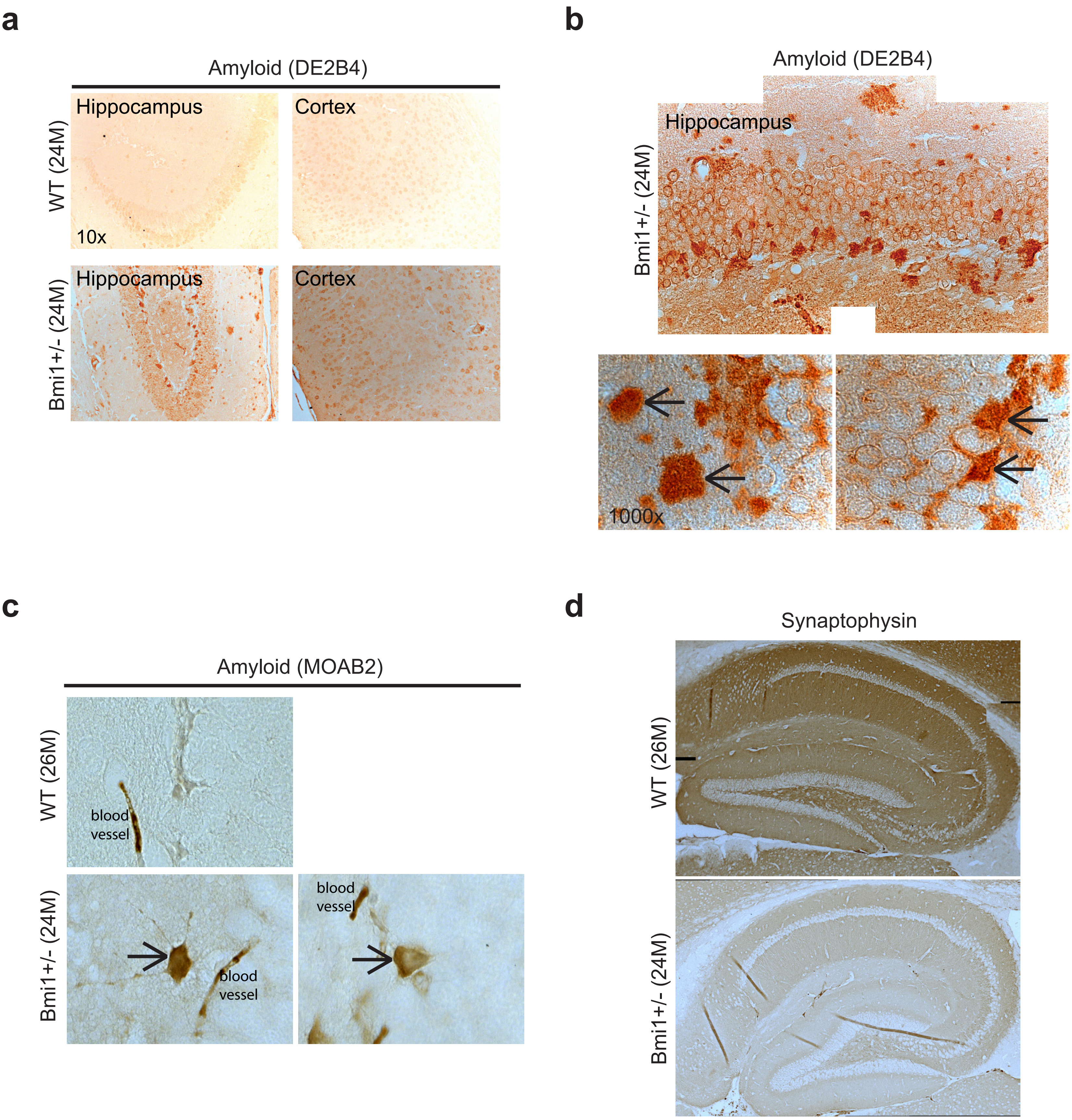
a
Amyloid (DE2B4)
WT (24M)
Bmi1+/- (24M)
Hippocampus
Cortex
10x
b
Amyloid (DE2B4)
Bmi1+/- (24M)
Hippocampus
1000x
c
Amyloid (MOAB2)
WT (26M)
Bmi1+/- (24M)
blood vessel
d
Synaptophysin
WT (26M)
Bmi1+/- (24M)

Figure S3

El Hajjar et al. 2018

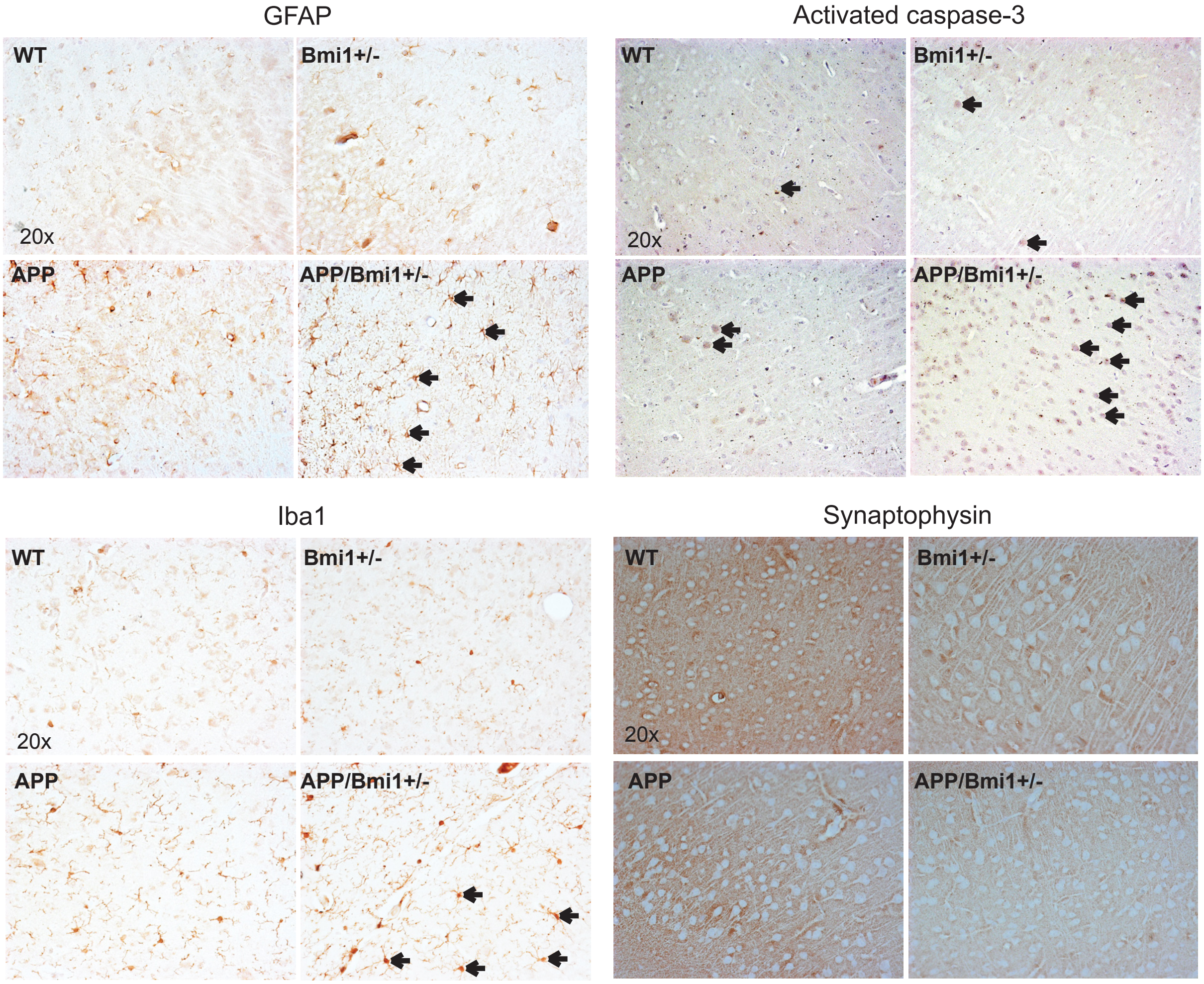

Figure S4

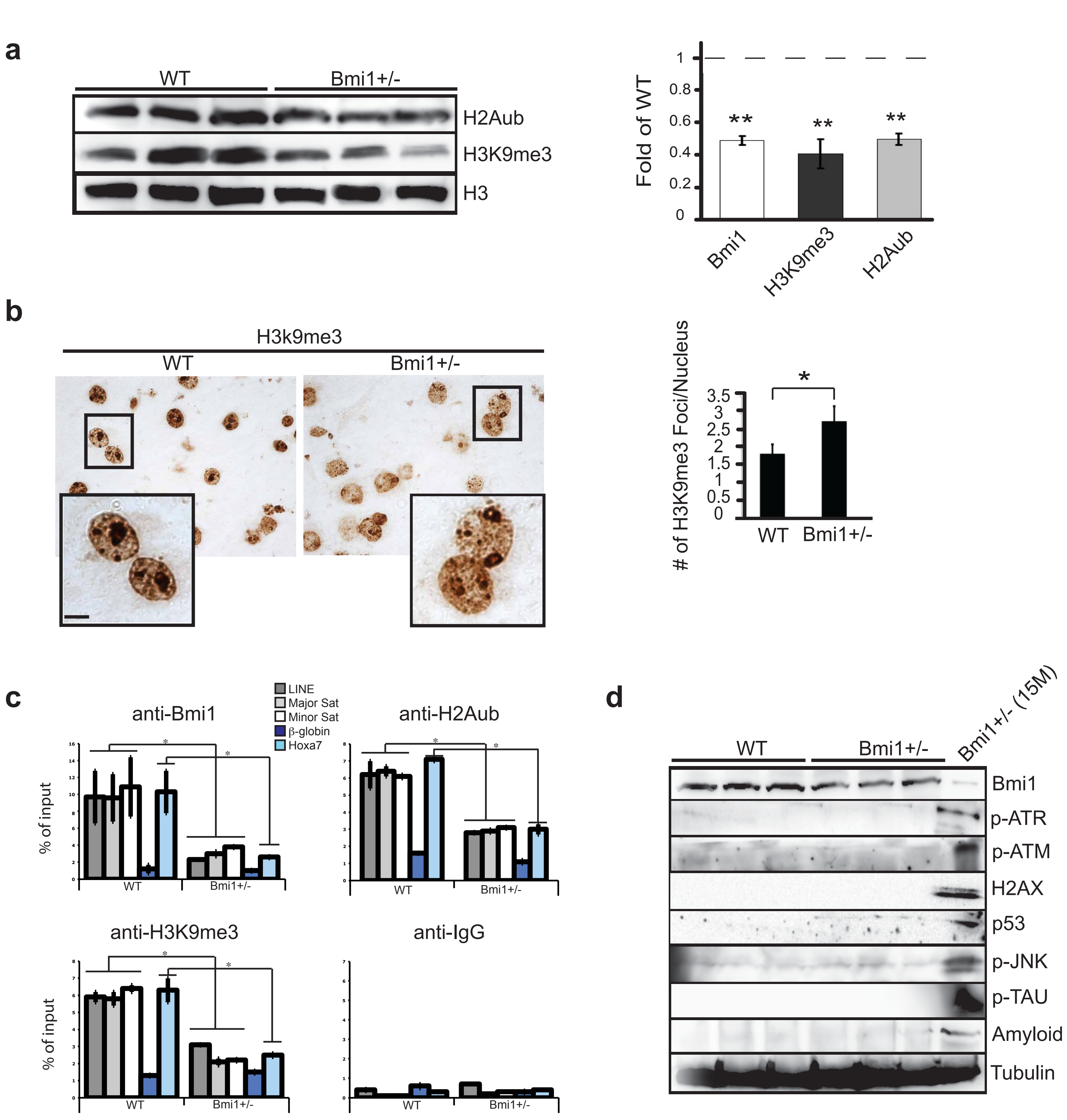
a
WT
Bmi1+/-
H2Aub
H3K9me3
H3
Fold of WT
1
0.8
0.6
0.4
0.2
0
**
**
**
Bmi1
H3K9me3
H2Aub
b
H3k9me3
WT
Bmi1+/-
# of H3K9me3 Foci/Nucleus
3.5
3
2.5
2
1.5
1
0.5
0
*
WT
Bmi1+/-
c
anti-Bmi1
LINE
Major Sat
Minor Sat
β-globin
Hoxa7
anti-H2Aub
% of input
WT
Bmi1+/-
anti-H3K9me3
anti-IgG
d
Bmi1+/- (15M)
WT
Bmi1+/-
Bmi1
p-ATR
p-ATM
H2AX
p53
p-JNK
p-TAU
Amyloid
Tubulin

Figure S5

Western blot and ChIP samples

	DH-Id # of samples	Sex	Age (yrs)	Median Age (yrs)
Frontal Cortex (Frozen)				
**Old controls**	428	M	89	**87.3**
	488	F	86	
	616	F	86	
	727	M	87	
	881	M	85	
	1487	F	91	
**LOAD**	999	F	87	**87.2**
	1018	M	88	
	1073	M	85	
	1127	M	88	
	1157	F	85	
	1599	F	90	
Hippocampus (Frozen)				
**Young controls**	1388	M	57	**59**
	1705	M	61	
	1722	M	59	
**EOAD**	1214	M	64	**61.3**
	410	M	63	
	1201	M	63	
	1147	F	55	

IHC samples

Frontal Cortex, (Fixed)	HMR-Id # of samples	Sex	Age (yrs)	Median Age (yrs)

**Old controls**	12c00024	M	90	**81.8**
	10c00052	M	72	
	10c00020	M	82	
**LOAD**	10c00088	M	75	**84**
	10c00042	M	89	
	10c00030	M	81	

Table S1. Human brain samples used in this study
Late-onset Alzheimer's disease (LOAD) and early-onset Alzheimer's disease (EOAD) samples were all confirmed to present "classical" Alzheimer's disease hallmarks by neuropathology. Old elderly and young controls were also confirmed as non-demented controls by neuropathology.
